# Profiling of polar urine metabolite extracts from Chinese colorectal cancer patients to screen for potential diagnostic and adverse-effect biomarkers

**DOI:** 10.7150/jca.47631

**Published:** 2020-10-08

**Authors:** Yi Deng, Houshan Yao, Wei Chen, Hua Wei, Xinxing Li, Feng Zhang, Shouhong Gao, Huan Man, Jing Chen, Xia Tao, Mingming Li, Wansheng Chen

**Affiliations:** 1 Department of Pharmacy, Changzheng Hospital, Secondary Military Medical University, Shanghai, China, 200003.; 2 Department of Surgery, Changzheng Hospital, Secondary Military Medical University, Shanghai, China, 200003.; 3 College of Chemical and Biological Engineering, Yichun University, Jiangxi Province, China, 336000.; 4 Research and Development Center of Chinese Medicine Resources and Biotechnology, Shanghai University of Traditional Chinese Medicine, Shanghai, China, 201203.

**Keywords:** colorectal cancer, UHPLC-Q-TOF-MS, untargeted metabolomics, capecitabine, adverse effect

## Abstract

**Background:** Metabolomics has demonstrated its potential in the early diagnosis, drug safety evaluation and personalized toxicology research of various cancers.

**Objectives:** We aim to screen for potential diagnostic and capecitabine-related adverse effect (CRAE) biomarkers from urinary endogenous metabolites in Chinese colorectal cancer (CRC) patients.

**Methods:** The metabolic profiles of 139 CRC patients and 50 non-neoplastic controls were analyzed using ultra-high-performance liquid chromatography combined with quadrupole time-of-flight mass spectrometry.

**Results:** There were 41 metabolites identified between the CRC patients and the non-neoplastic controls, and 19 metabolites were identified between CRC patients with and without CRAE. Based on these identified metabolites, bioinformatic analysis and prediction model construction were completed. Most of these differential metabolites have important roles in cell proliferation and differentiation and the immune system. Based on binary logistic regression, a CRC prediction model, composed of 3-methylhistidine, N-heptanoylglycine, N^1^,N^12^-diacetylspermine and hippurate, was established, with an area under curve (AUC) of 0.980 (95% CI: 0.953-1.000; sensitivity: 94.3%; specificity: 92.0%) in the training set, and an AUC of 0.968 (95% CI: 0.933-1.000; sensitivity: 89.9%; specificity: 92.0%) in the testing set. In addition, methionine and 4-pyridoxic acid can be combined to predict hand foot syndrome, with an AUC of 0.884; ubiquinone-1 and 4-pyridoxic acid can be combined to predict anemia, with an AUC of 0.889; and 5-acetamidovalerate and 3,4-methylenesebacic acid can be combined to predict neutropenia, with an AUC of 0.882.

**Conclusion:** The profiling of urine polar metabolites has great potential in the early detection of CRC and the prediction of CRAE.

## Introduction

Colorectal cancer (CRC) is one of the most common malignancies worldwide, with an estimated 1.4 million new diagnosed cases and 693,900 death cases in 2012 1. Over the last few years, with the changes of risk factors and the introduction of early screening, the incidence rates and death rates of CRC have declined in the United States 2. However, the incidence and mortality rates are increasing rapidly in developing countries like China 3. Although the 5-year survival rate of stage I patients can reach nearly 90%, the rate of stage IV patients is only 12% 4. Thus, the early detection of CRC is of central importance to improve overall survival rates. Colonoscopy, which is currently the gold standard for CRC diagnosis, is invasive and uncomfortable 5. Computed tomography colonography (CTC) is an accurate and reliable diagnostic technique, but its high cost has always been a problem. Fecal occult blood testing (FOBT), as well as other noninvasive and inexpensive plasma biomarkers, such as carcinoembryonic antigen (CEA), carbohydrate antigen 19-9 (CA19-9) and *SEPT9* gene methylation, are the main screening methods. However, their sensitivity and specificity are relatively poor, and screening with these biomarkers can easily miss asymptomatic patients. Therefore, simple, noninvasive, highly sensitive and specific biomarkers are urgently required for the early diagnosis of CRC.

Metabolomics, which is the comprehensive study of low molecular weight metabolites and potentially offers phenotypic information not captured by genetic profiling, has become the focus of modern systematic biology 6. It has demonstrated its potential in the early diagnosis, drug safety evaluation and personalized toxicology research related to various cancers 6,7,8, including CRC 9. To date, by identifying the metabolic profiles in blood, urine, stool and tissue samples between CRC patients and healthy counterparts, significant variations have been revealed, and a number of candidate biomarkers identified 9. However, none of these biomarkers have entered into clinical practice.

There has also been research on the application of metabolomics for prediction of drug-induced adverse effects (AEs) 10, 11, 12, 13, 14. Studies of metabolic biomarkers of oncology are relatively rare. According to the National Comprehensive Cancer Network (NCCN) guideline (2016), the first-line CAPEOX protocol, containing capecitabine and oxaliplatin, is usually used for both early postoperative adjuvant chemotherapy and advanced palliative chemotherapy. However, AEs remain the major limitation in treatment, especially for bone marrow suppression (BMS) and hand foot syndrome (HFS). Both BMS and HFS were selected as AEs for analyses in this study, since our previous clinical observation and literature research showed that these two AEs have the highest incidence rates 15.

In this article, a urinary metabolomics study was conducted on a cohort of CRC patients (n = 139) and non-neoplastic control subjects (n=50) using ultra-high-performance liquid chromatography combined with quadrupole time-of-flight mass spectrometry (UHPLC-Q-TOF-MS). The purpose of this study is to screen endogenous metabolite biomarkers, and to establish prediction models for CRC diagnosis and capecitabine-related AEs (CRAEs).

## Methods

### Chemicals and reagents

Acetonitrile, methanol and isopropanol (HPLC grade) were purchased from Merck (Darmstadt, Germany). Chloroform, formic acid, ammonium acetate and other solvents (analytical grade) were purchased from Tedia (Fairfield, CT, USA). Internal standard (L-2-chlorophenylalanine) was purchased from Sigma Aldrich (St. Louis, MO, USA).

### Clinical samples

The 139 patients were 36-87 years old and diagnosed with CRC (72 colon cancers and 67 rectal cancers). They were selected from a registered ongoing clinical trial at Shanghai Changzheng Hospital (code at www.clinicaltrials.gov, NCT03030508) from June 2016 to June 2017. The ethical approval for the study was granted by Shanghai Changzheng Hospital Biomedical Research Ethics Committee (approval number: 2016SL007). Recruited subjects in CRC patients were (1) over 18 years old and (2) diagnosed with CRC by biopsy examination. Patients with any preoperative anti-neoplastic medication were excluded. Clinical information was obtained from the hospital and provided in Table S1. The 50 non-neoplastic controls were aged 47-89 years. They were without any known inflammatory condition or gastrointestinal tract disorders, and were enrolled after a routine physical examination. The age and sex of the controls were equivalent to those of the CRC patients (Table S1). Prior to sample collection, a written informed consent was obtained from each patient.

To ensure the effectiveness of the CRC diagnostic model, all samples were randomly divided into a training set and a test set with a ratio of 1:1 using Excel (Microsoft, USA). The two sets were well-matched between CRC patients and control groups in age and sex (Table S1). Among the 139 CRC patients, 43 had received capecitabine-based adjuvant chemotherapy. For these patients, HFS and BMS (including anemia, neutropenia and thrombocytovpenia) were followed-up and graded according to Common Terminology Criteria for Adverse Events (Version 4.0) (Table S2) (2010). These patients were divided into AE and no-AE groups, respectively. Student's *t* test showed no significant difference in age and chemotherapy cycle between the two groups. Chi-square test showed no difference in sex, and Mann-Whitney test showed no difference in the pathological stage, between CRAE and no-CRAE groups (Table S3).

All urine samples were collected from the Department of General Surgery in Shanghai Changzheng Hospital. A 12-mL urine sample was collected into a Falcon tube 1-3 days before surgery with an empty stomach, followed by adding 1 mL of protease inhibitor mixture (0.4 mL of 100 mM NaN_3_, 0.6 mL of 10 mM phenylmethylsulfonyl fluoride and 50: l of 1 mM leupeptin) 16. Then, the samples were stored at -80°C.

### Sample preparation

Since urinary metabolites were concentrated in polar metabolites and contained only few non-polar metabolites, we focused on polar metabolites in this study by separating the polar content and using a separation column specialized for polar metabolites. A volume of 10 μL of urine from each sample was mixed and used as the quality control (QC). The QC sample was made for testing the instrument state, equilibrating the UHPLC-Q-TOF-MS system before sample injection and indicating system stability during the batch analyses 17. Subsequently, the polar metabolites were extracted from 200 μL of urine sample or QC sample with 800 μL of chloroform/methanol (2:1, v/v) spiked with 0.2 μg/mL L-2-chlorophenylalanine as the internal standard in a fume hood. Then, the mixture was vortexed for 1 min and centrifuged at 15,000 × g for 10 min at 4°C to remove protein and split the polar (supernatant layer) and non-polar metabolites (lower phase). An aliquot of 300 μL from the supernatant was transferred to a 1.5-mL EP tube, mixed with 900 μL of methanol and centrifuged at 12,000 g for 10 min at 4°C. Next, 900 μL of the supernatant was lyophilized. The lyophilized sample was resuspended in 900 μL of acetonitrile, and stored at -80°C. Stored samples were thawed at 4°C before analysis. Finally, 200 μL of the solution was transferred to a plastic insert within a sampler bottle for injection in the UHPLC-Q-TOF-MS system.

### UHPLC-Q-TOF-MS analysis

Sample analysis was performed on an Agilent 1290 ultra-high-performance liquid chromatography system (Agilent Technologies, Santa Clara, CA, USA) coupled with an Agilent 6530 Accurate-Mass Q-TOF LC/MS system (Agilent Technologies) in positive Dual Agilent Jet Stream Electrospray Ionization (Dual AJS ESI) mode (Agilent Technologies). The mobile phases A and B were water with 0.1% v/v formic acid and acetonitrile with 0.1% v/v formic acid, respectively. The column was a 2.1 × 100 mm, 3.5 μm, HSS T3 column (Waters, Manchester, UK) and the temperature was kept at 30°C. The gradient started with 5% B, increased to 20% at 6 min, 50% at 9 min, 95% at 13 min, 100% at 15 min, followed by a post-run of 5 min. The flow rate was maintained at 0.4 mL/min. The injection volume was 3 μL. The capillary voltage was 3500 V, and the nozzle voltage was 500 V. The gas temperature was set at 300°C with a gas flow of 11 L/min and nebulizer pressure of 35 psi, and a sheath gas temperature of 300°C with a sheath gas flow of 11 L/min. For MS acquisition, centroid data were acquired from 100 to 1100 m/z at 0.5-s intervals. For MS/MS acquisition, data were acquired at 0.33-s intervals with collision energy 0, 10, 20 and 40 eV. A reference solution (m/z 121.0509 and m/z 922.0098) was used to correct small mass drifts during the acquisition 17. The QC samples were injected at the beginning of the run and after every eight samples during sequence analysis to assess the analytical performance 18.

### Data analysis

The acquired MS data were analyzed using the Profinder program (Version b8.0, Agilent Technologies). After integration and alignment, a list of spectral features was obtained with the retention time (RT), m/z and spectral area by recursive feature extraction. The spectral features generated by the internal standard, noise and column bleed were removed from the dataset. Then, the integration results were manually checked before they were transferred to the Mass Profiler Professional program (Agilent Technologies) for subsequent analysis. The background and non-biologically relevant information were eliminated according to the 80% rule 19, which means only spectral features with a frequency ≥ 80% in the CRC patient or control groups were kept. Then, these spectral features were normalized using the sum intensity of each feature in each sample.

Soft Independent Modelling by Class Analogy 14.0 (SIMCA, Umetrics AB, Umeå, Sweden) and SPSS version 17.0 (SPSS Inc., Chicago, IL, USA) were used for further analyses. A *P*-value of less than 0.05 was considered significant. Principal Component Analysis (PCA) was applied to examine data distribution, and for a comprehensive understanding of the metabolic profile. Orthogonal Partial Least Squares Discriminant Analysis (OPLS-DA) was carried out to focus on clustering information and visualize the metabolic alterations. Multivariate statistical analysis in SIMCA 14.0 was used to analyze the complex metabolomics. Criteria for potential biomarkers were a coefficient of variation (CV) < 30% in QC samples. The affected metabolic pathways were examined by Metabolic Sets Enrichment Analysis (MSEA) in MetaboAnalyst 4.0. Student's *t* test was performed between the two groups to select biomarker candidates. Spectral features with a low *P*-value (< 0.05) and a high fold of change (FOC ≥ 2) in Student's *t* test, or with the value of variable importance in the projection (VIP) more than 1 in the OPLS-DA model were added to the candidate list for further metabolite identification. These metabolites were identified by an integrated method which included comparing to commercially approached standards and the web-based spectrum databases such as the Human Metabolite database (http://www.hmdb.ca/) and METLIN (http://metlin.scripps.edu/) 21,22.

Then, binary logistic regression was applied to combine several variables into a multivariable, using a stepwise variable selection method. Receiver operating characteristic (ROC) curve analysis was performed to evaluate the predictive ability of each identified metabolite and the combinational multivariable.

## Results

### Urinary metabolic profiling

Typical total ion current (TIC) chromatograms of the metabolic profiles are shown in Figure S1. All pooled QC samples were used to monitor the system stability and data reliability for peak intensity (<30% CV) and RT (<20% CV). After manually checking, the metabolomics data revealed 1114 peaks of polar compounds detected by Q-TOF LC/MS and 583 peaks were screened by the 80% rule.

**Figure 1:**
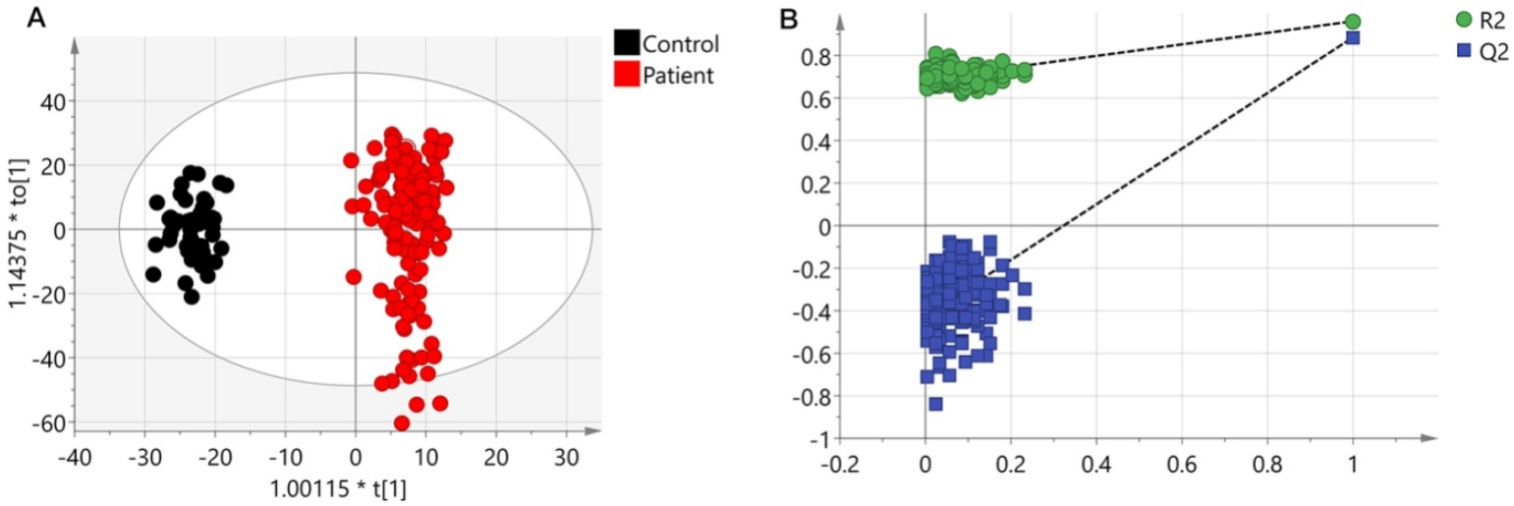
** Results from UHPLC-Q-TOF-MS.** (A) An OPLS-DA scores plot discriminating urine samples from CRC patients (black boxes) and non-neoplastic controls (red dots) using UHPLC-Q-TOF-MS positive ion model analysis. (B) The chance permutation test at 999 times strongly supported the validity of the established OPLS-DA model.

A PCA model (two components, R^2^X_cum_ = 0.366 and Q^2^_cum_ = 0.338) with unit variance (UV) scaling and an OPLS-DA model (one predictive component and three orthogonal components, R^2^X_cum_ = 0.306, R^2^Y_cum_ = 0.963 and Q^2^_cum_ = 0.89) based on Pareto Variance (Par) scaling were established using the 570 spectral features. The QC samples showed tight clustering but separation between control and patients was not clear in the PCA (Figure S2). This separation was more obvious in the OPLS-DA model (Figure 1A). A 999-time permutation test was performed to evaluate the PLS-DA model. The R^2^Y- and Q^2^-intercepts were 0.692 and 0.412, respectively (Figure 1B). The validation plots from permutation tests strongly supported the validity of the established OPLS-DA model because all permuted R^2^ and Q^2^ values on the left were lower than the original point on the right, and the Q^2^ regression line in blue had a negative intercept.

Different metabolites between controls and CRC patients were identified using Student's *t* test (*P* ≤ 0.05 and FOC ≥ 2), or VIP ≥ 1 in the OPLS-DA model. A total of 281 compounds were screened and 41 metabolites were identified by comparing metabolomic databases (Table 1, Table S4). Subsequently, 19 differential identified metabolites were found to be related to CRAE based on Mann-Whitney tests (Table 2).

### Metabolic pathway analyses

In order to understand the significant differences in the metabolic networks between the CRC patients and the controls, the 41 CRC related metabolites identified were submitted to the CPDB website (http://cpdb.molgen.mpg.de/) for metabolic pathway enrichment analysis. This analysis was also repeated for the 19 CRAE related metabolites. The MSEA results are shown in Tables 3 and 4. There were 15 CRC related and 10 CRAE related metabolic pathways enriched. For the CRC related metabolic pathways, majority of them are related to the synthesis and catabolism of some of the basic metabolites such as basic carboxylic acid and amino acids. These metabolic pathways include glucose homeostasis, conjugation of carboxylic acids, amino acid conjugation and etc. Some of these changes may be related to abnormal DNA synthesis, since one carbon metabolism and related pathways and folate metabolism were also enriched. Beside these, vitamin B12 metabolism was also found related to CRC, this may indicate that abnormal in inflammation response or immune system may also be related to the susceptibility of CRC.

On the other hand, CRAE related metabolic pathways are similar to the CRC related metabolic pathways. Pathways including conjugation of carboxylic acids, amino acid conjugation, glucose homeostasis indicate altered fundamental synthesis and catabolism of some of the basic metabolites. Pathway such as B12 metabolism indicates that abnormal in inflammation response or immune system may also be related to the susceptibility of CRAE as well.

### Construction and validation of a diagnostic biomarker metabolite system

**Figure 2:**
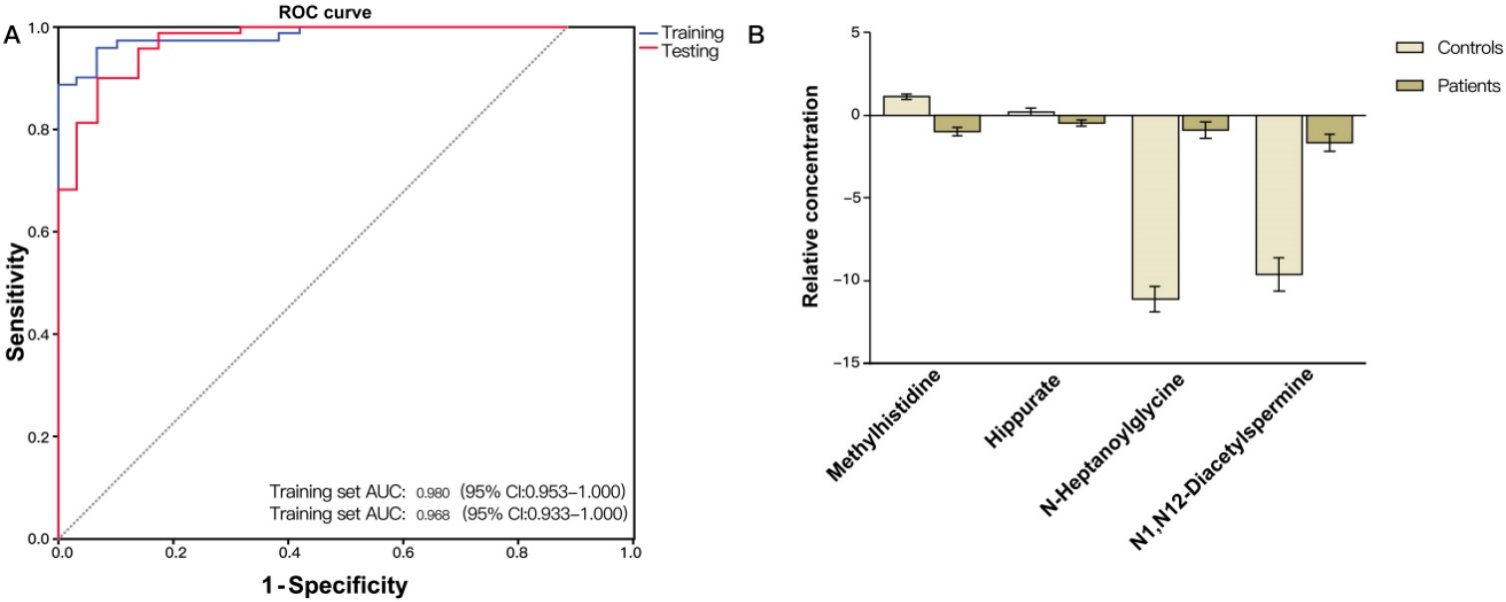
(A) ROC curve analysis of the ability of urinary metabolites including methylhistidine, N-heptanoylglycine, N^1^,N^12^-diacetylspermine and hippurate to discriminate between CRC patients and non-neoplastic controls. The area under the curve (AUC) was 0.980 (95% CI: 0.953-1.000) for the training set (blue line), and 0.968 (95% CI: 0.933-1.000) for the testing set (red line). (B) Bar charts of the mean concentrations of methylhistidine, N-heptanoylglycine, N^1^,N^12^-diacetylspermine and hippurate between CRC patients and non-neoplastic controls.

**Figure 3:**
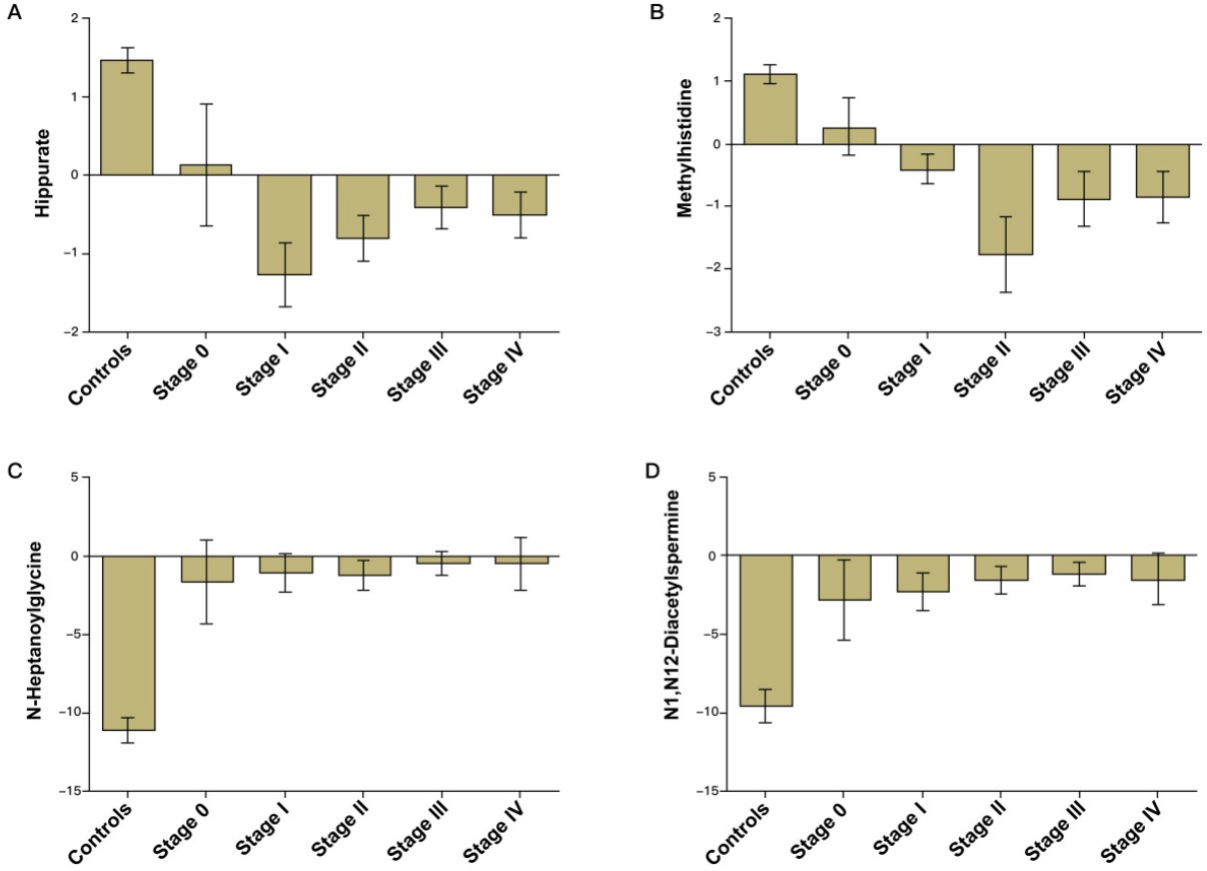
Bar charts of the mean concentrations of methylhistidine, N-heptanoylglycine, N^1^,N^12^-diacetylspermine and hippurate in urine samples of CRC patients of different stages and non-neoplastic controls.

Validation of the CRC diagnostic model was performed by randomly choosing 50% of the samples to create a training-test set. The two sets were well-matched between CRC patients and control groups in age and sex (Table S1). A training set was used to evaluate the validation and predictive ability of identified metabolites to construct a diagnosis marker system for potential clinical application. Based on their high FOC, AUC and VIP values, four metabolites were selected as a panel of candidate markers: methylhistidine, N-heptanoylglycine, N^1^, N^12^-diacetylspermine and hippurate. A binary logistic regression model was applied to combine the four variables into a multivariable model. The ROC curve showed that the training set had an AUC value of 0.980 (95% CI: 0.953-1.000; sensitivity: 94.3%; specificity: 92.0%), and the testing set had an AUC value of 0.968 (95% CI: 0.933-1.000; sensitivity: 89.9%; specificity: 92.0%) (Figure 2A). The relative concentrations of these metabolites in urine samples of CRC patients and non-neoplastic controls are shown in Figure 2B. Spearman's rank correlation coefficient test showed that the concentrations of these metabolites were not related to the pathological stages (*P* > 0.05) (Figure 3).

### Construction of prediction models for CRAEs

**Figure 4:**
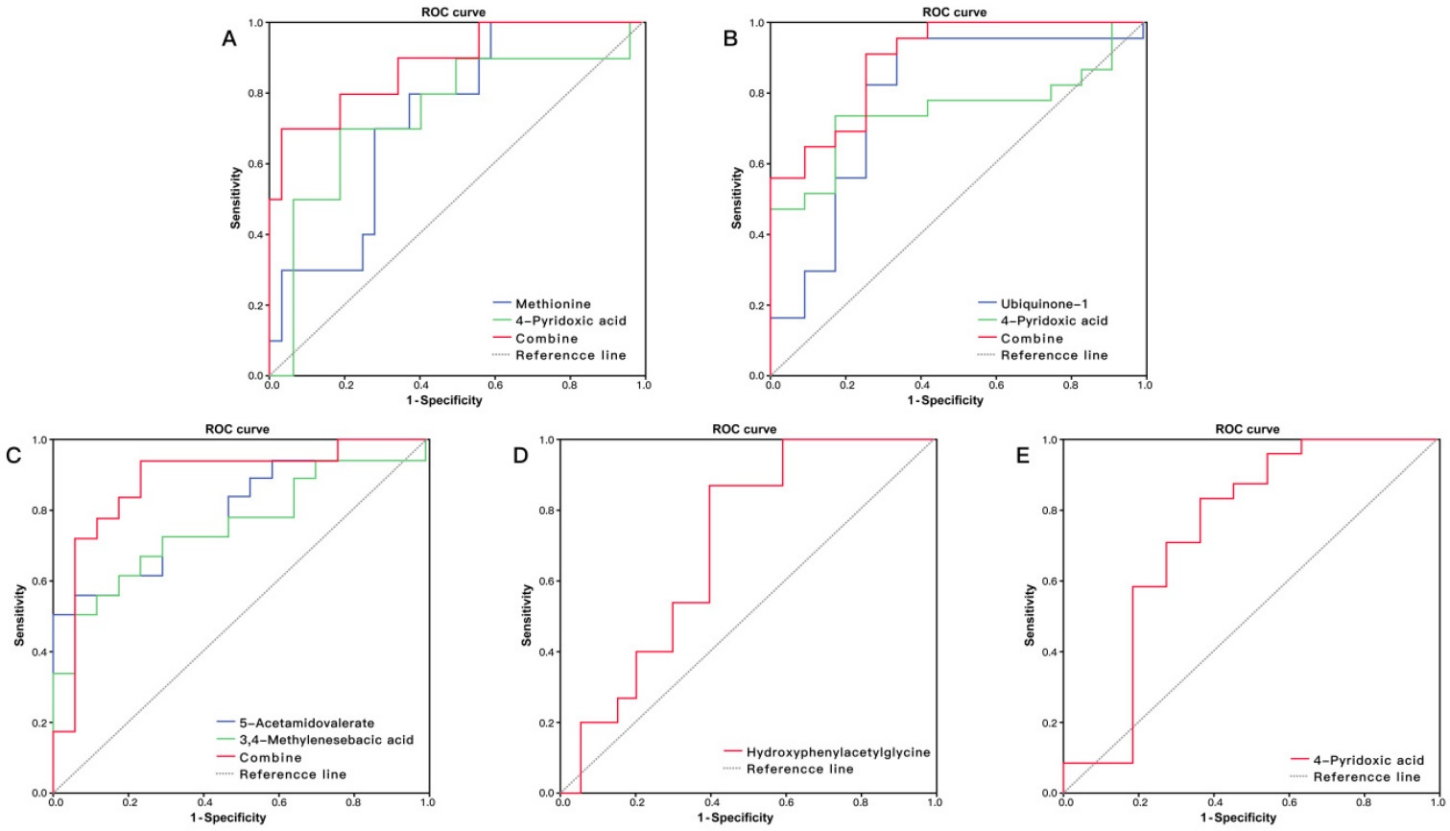
(A) ROC curve analysis of the ability of urinary methionine and 4-pyridoxic acid to predict hand foot syndrome (HFS). The area under the curve (AUC) was 0.884 (95% CI: 0.746-1.000). (B) ROC curve analysis of the ability of urinary ubiquinone-1 and 4-pyridoxic acid to predict anemia. The AUC was 0.889 (95% CI: 0.786-1.000). (C) ROC curve analysis of the ability of 5-acetamidovalerate and 3,4-methylenesebacic acid to predict neutropenia. The AUC was 0.882 (95% CI: 0.752-1.000). (D) ROC curve analysis of hydroxyphenylacetylglycine to predict thrombocytopenia (AUC = 0.700, 95% CI: 0.526-0.874). (E) ROC curve analysis of 4-pyridoxic acid to predict overall bone marrow suppression (AUC = 0.739, 95% CI: 0.529-0.948).

The ROC curve analysis showed that five metabolites had potential to predict HFS (*P* < 0.05, AUC > 0.7): methionine, 5-acetamidovalerate, N^1^, N^12^-diacetylspermine, 4-pyridoxic acid and indolylacryloylglycine (Table 2). Thirteen metabolites had predictive ability for anemia: 5-acetamidovalerate, methylhistidine, N-acetylaminooctanoic acid, indoleacetic acid, 1-methyl-2-nonyl-4(1H)-quinolinone, 3,4-methylenesebacic acid, hippurate, aspartylphenylalanine, phenylacetylglutamine, ubiquinone-1, 4-pyridoxic acid, creatinine and Indoxyl (*P*<0.05, AUC>0.7). Hydroxyphenylacetylglycine had potential for predicting thrombocytopenia (AUC = 0.700, 95% CI: 0.526-0.874) (Figure 4D), and 4-pyridoxic acid had potential for prediction of overall BMS (AUC = 0.739, 95% CI: 0.529-0.948) (Figure 4E).

Logistic regression showed that the combination of methionine and 4-pyridoxic acid had high discriminatory ability for HFS with AUC = 0.884 (Figure 4A), and the combination of ubiquinone-1 and 4-pyridoxic acid had an obvious predicting advantage over a metabolite for anemia, with AUC = 0.889 (Figure 4B). The combination of 5-acetamidovalerate and 3,4-methylenesebacic acid showed better predictive performance than a single metabolite, with AUC = 0.882 (Figure 4C).

## Discussion

### Biochemical functions of CRC-related differential metabolites

Like most other cancers, CRC has an uncontrolled cell cycle progression, rapid growth rate, loss of contact inhibition, increased glycolysis and a triggered host immunological response. As a result, CRC patients had a differential plasma metabolic profiling compared to the non-neoplastic controls. Therefore, metabolic analyses and metabolites can indicate potential diagnostic markers and help to reveal the underlying mechanisms of cancer development and drug metabolism 9, 21, 22, 23, 24, 25, 26, 27, 28.

Some of the differential compounds identified in the CRC patients here might result from the rapid metabolic rate and altered energy metabolites of cancer cells. The CRC patients showed abnormal Glucose Homeostasis. The significantly decreased urine glucose level (Table 1) is consistent with one previously published study 29. This might indicate elevated glucose consumption in CRC patients compared to the controls.

The N^1^, N^12^-diacetylspermine is a constituent polyamine in human urine 30. Polyamines are indispensable in cell growth, gene expression and cell proliferation 31. Rapidly growing cells, such as cancer cells, generally have increased intracellular polyamine levels and actively metabolize polyamines. An elevated N^1^, N^12^-diacetylspermine level may indicate rapid proliferation of cancer cells themselves 32, and has been reported as a more sensitive biomarker than CEA, CA19-9 or CA15-3 for CRC diagnosis at early stages 30, 31, 32, 33.

Methionine is an essential amino acid, involved in the pathways for glucose homeostasis, vitamin B12 metabolism, amino acid metabolism, central carbon metabolism in cancer, one carbon metabolism, and folate metabolism. Methionine metabolism is relevant for cancer pathogenesis including methylation reactions, redox maintenance, polyamine synthesis and coupling to folate metabolism to coordinate nucleotide and redox status 34. One carbon metabolism and Folate Metabolism are involved in regulation of the genetic process from DNA synthesis to cell migration, proliferation, differentiation and apoptosis 35, 36. Down-regulation of methionine may indicate increased protein biosynthesis in cancer cells. Since methionine also plays a role in DNA methylation by providing methyl groups, overconsumption of methionine for protein biosynthesis may cause overall DNA hypomethylation, which could reduce DNA stability and trigger CRC development 37, 38. Compared to the non-neoplastic controls, CRC patients normally have lower methionine levels both in serum 28 and urine 25, but higher levels in tissues 21. High plasma concentration of methionine is a marker of low CRC risk 39. Despite the role in protein biosynthesis, down-regulated methionine level in CRC may indicate a low level of auto-inflammation, which is closely related to antioxidant defenses in some organs 40, 41, 42.

Methylhistidine is a result of excessive protein catabolism, and down-regulated methylhistidine may also indicate overall increased protein biosynthesis in CRC patients. A study of 63 CRC patients' urine metabolites also supported down-regulated histamine metabolism 43; however, one study showed that neither urinary 1-methylhistidine nor 3-methylhistidine was associated with colorectal adenoma in a single urine sample, but worthy of further investigation in considering multiple urine samples 44. Similarly, 5-acetamidovalerate is a product of lysine catabolism 45. Both alanylasparagine and glutamylproline are dipeptides. They are the products of incomplete catabolism of large proteins. All of these metabolites are down-regulated in CRC patients compared with controls. This indicates the protein thesis was elevated by CRC.

Phenylacetylglutamine (PAG) is a common metabolite of fatty acids with low abundance. It is a colonic microbial metabolite from amino acid fermentation, generated from glutamine conjugation of phenylacetic acid almost exclusively derived from the microbial conservation of phenylalanine, constituting phenylacetate metabolism, which provides a route that facilitates the excretion of nitrogen for patients with urea-cycle defects. Compared to the controls, CRC patients had lower PAG in this study, which may derive from down-regulated phenylalanine metabolism and glutamine metabolism related to gut flora metabolism 25.

Hippurate and its metabolite hydroxyhippurate are normal constituents of endogenous urinary metabolites, generated from microbial degradation of certain dietary components including phenylalanine. As a downstream product of phenylalanine, a decreased level of hippurate also indicates down-regulated phenylalanine metabolism 25.

Other differential compounds identified might indicate the different inflammatory response and the degradation of fatty acids between CRC patients and non-neoplastic controls. N-Heptanoylglycine contains a C-7 fatty acid group as its acyl moiety, which is a minor metabolite of dietary fatty acid. Elevated levels of certain acylglycines in urine and blood may indicate patients with various fatty acid oxidation disorders.

The lower serum level of coenzyme Q (CoQ) was reported and speculated to be associated with CRC progression 28. Ubiquinone-1 is an intermediate in CoQ synthesis and could act as an antioxidant. The CoQ can suppress fat-induced colon carcinogenesis as an antioxidant 46 and the level of CoQ was also reported to be negatively correlated with redox status 47.

As the main catabolic product of vitamin B6, urinary 4-pyridoxic acid level is significantly associated with the circulating level of vitamin B6 48. Vitamin B6 itself is only modestly associated with inflammation; however, the PAr ratio [4-pyridoxic acid/ (pyridoxal + pyridoxal 5′-phosphate)] is an indicator of vitamin B6 catabolism during inflammation, which is also a risk factor for carcinogenesis 49, 50.

Here, the elevated inflammatory status in CRC patients is consistent with the changes of metabolites arising from bacterial protein catabolism, particularly the tryptophan metabolism 51, 52, 53. Tryptophan and its bacterial metabolites play various roles in the balance between immune tolerance and gut microbiota maintenance. The relationship between bacterial tryptophan metabolism and immune response has been described in detail by a recent review 53. Indole is formed in intestines from tryptophan, and then it is transferred into indoxyl in the liver 54, 55. The serum concentration of indoxyl has been found to decrease in azoxymethane/dextran sodium sulfate (AM/DSS)-induced colon cancer mice 56. In line with this result, this decreased urinary level was observed in our CRC patients.

Tryptophan can be converted into indole pyruvic acid by aromatic amino acid aminotransferase, which can be further converted into indole acetaldehyde, and then into indole acetic acids (e.g. indole-3-acetic acid [IAA]). Its level in CRC tissues was found to be significantly decreased compared with the normal tissues 57. Consistently, the urinary level of tryptophan in our CRC patients was also decreased. Pyruvic acid can also be converted into indole acrylic acid, and then finally into indolylacryloylglycine (IAcrGly) through a few enzyme-controlled steps. IAcrGly is one of the physiological components in urine. It was hypothesized that abnormal gut flora could promote the conversion of tryptophan to indolyl propionic acid, which could cause an increased IAcrGly level in urine 58, 59. The trans-verse situation might be true for patients with bladder or CRC. It has been qualified as a part of model of bladder cancer grading distinction. As the increase of pathologic stage malignant degree of gallbladder, the concentration of IAcrGly decreased in high-grade bladder cancer compared with low-grade bladder cancer 60. Herein, a significant decrease of urinary concentration of IAcrGly in CRC patients was also found. Decreased IAcrGly alone is also a sign of elevated inflammation response, since it is closely associated with introduced oxidative damage by adulterants and elevated oxidative damage is one of the CRC's characteristics 61, 62. Taken together, the urinary levels of IAA, indoxyl, and IAcrGly were all down-regulated in our CRC patients, which suggested a suppressed production of indole pyruvic acid and its derivatives. This may also be contributed by overexpressed indoleamine 2,3-dioxygenase that depletes tryptophan in CRC 63. Metabolites of indole pyruvic acids including IAA, indoxyl, and IAcrGly are ligands to aryl hydrocarbon receptor (AHR), a transcriptional regulator for intestinal innate immunity and inflammation in the colitis-associated tumorigenesis. These metabolites are beneficial for colon by suppressing inflammation and carcinogenesis 64, 65. The down-regulated IAA, indoxyl, and IAcrGly in our CRC patients compared with the controls may indicate the elevated inflammation response and induced carcinogenesis.

It is worth mentioning that another bacterial tryptophan metabolite N-acetyltryptophan (NAT) was found to be up-regulated in our CRC patients compared with the controls. Its upregulation was also reported in the case of compromised gut microbiota 66, 67. NAT can prevent protein molecules from oxidative degradation by scavenging oxygen 68. In this study, the up-regulation of NAT further confirmed the development of imbalanced bacteria in CRC, but its physiological function in CRC still needs to be investigated.

In conclusion, based on the urinary metabolomic profile, the CRC patients showed elevated protein metabolism rate, induced inflammation response, and possibly increased energy consumption, compared with the controls.

### Biochemical functions of CRAE-related differential metabolites

The pharmacological process of capecitabine has been fully reviewed in both *in vivo* and *in vitro* studies. DNA polymorphism 69, 70, 71, DNA methylation differences 72 and pharmacokinetic measurements 73 that could reflect the pharmacological process of capecitabine have been used to predict CRAE. Some of them have already been proved by prospective clinical research 71. However, the pharmacological process only determines the local level of capecitabine-related cytotoxicity. In addition to this, how DNA replication, cellular proliferation, cellular apoptosis and immunology systems of normal tissue cells respond to the cytotoxicity may also contribute to the susceptibility to CRAE.

According to the literature 74, 75 and our ongoing observational clinical trial 15, BMS and HFS are the two most frequent CRAEs, which severely limit the usage of capecitabine. The BMS contains three sub-types of AEs: anemia, thrombocytopenia and neutropenia. The direct cause of BMS is suppressed blood cell formation, which is a multistep process that starts from differentiation of hematopoietic stem cells and ends with the formation of types of blood cells 76, 77. It is tightly regulated by signaling mediators, growth factor receptors and transcriptional factors involved in cell proliferation and differentiation 78. The direct cause of capecitabine-related-HFS is a type of inflammation response mediated by COX-2 over-expression in the palm and plantar 79. Therefore, differential metabolites related to cell proliferation, differentiation and immunological response might be potential markers of CRAE.

To date, there are only a few published literatures that apply metabolomics to investigate markers for CRAE. One previous study showed that higher levels of low-density lipoprotein prior to treatment could predict higher grade toxicity for advanced CRC patients who received single-agent capecitabine 80. Abnormally high level of low-density lipoprotein alone is a hazard factor for immunological response 81.

Consistent with our theory, levels of N^1^, N^12^-diacetylspermine were down-regulated in patients who developed HFS compared to those had not. This may indicate faulty DNA synthesis. In addition, a number of indicators and mediators of inflammation response were consistently altered in patients with CRAEs. These included up-regulated 4-pyridoxic acid and down-regulated methionine and methylhistidine. Interestingly, the differential inflammation responses were also revealed by metabolites from bacterial tryptophan catabolism. We observed relatively lower levels of IAA and indoxyl in CRC patients susceptible to anemia, and lower levels of IAcrGly in CRC patients susceptible to HFS. Since both IAA and IAcrGly can activate AHR that exert protective effects on autoimmune inflammation 82, 83, 84, 85, the urinary levels of which are mediated by tryptophan metabolism and gut microbiota, we speculate that the altered gut microbiota may also be an important factor for the susceptibility to CRAE. In summary, urinal metabolomics is affected by the health condition of individual, including proliferation, differentiation, and inflammation. It suggests that CRC patients who are susceptible to CRAEs may have faulty proliferation, differentiation, and induced inflammation.

### Summary and future directions

The main strength of this study is that it explored CRAE-related metabolites for the first time. A number of metabolites were identified and a potential CRAE predicting model was generated. We also identified CRC-related metabolites. Based on these metabolites, a diagnostic model was generated and verified.

However, several limitations of this study have to be mentioned and considered for future analysis. First, the sample population was small. Our patients were exclusively enrolled from one clinical center and the majority were from the south-east part of China. Second, although the patients and controls had equivalent age and sex, other intrinsic and environmental factors with possible influence were not assessed. Third, because of the small sample size of the CRC patients, internal replication was not used for CRAE prediction models. The reliability of these models will need to be tested using a larger population. Fourth, only positive results were compared with other positive results from the literature. Ideally, we should have also compared our results with other negative results; however, since not many studies report negative results, there is no symmetrical way to do this. Therefore, our results may be found to be negative by others. For example, in our study, methylhistidine was not associated with CRC, unlike the report by Cross et al. 44.

In summary, comparing CRC patients and non-neoplastic controls, and CRC patients with and without CRAEs, differential metabolites revealed changes in cell differentiation and immune response. We speculate that induced proliferation of cancer cells and altered immune response were associated with the specialized metabolic profile of CRC patients. However, faulty cell proliferation, cell differentiation, potential metabolic pathways and excessive immune response may make the CRC patients more susceptible to CRAEs.

## Conclusions

Based on urinary metabolic profiles, we identified a number of metabolic pathways associated with CRC and CRAE. Most of these differential metabolites have important roles in cell proliferation, differentiation and immune response. We also constructed a series of biomarker systems for CRC diagnosis and CRAE prediction.

## Supplementary Material

Supplementary figures and tables.

## Figures and Tables

**Table 1:** Identified metabolites related to colorectal cancer

No.	Metabolites	Formula	Mass	*t_R_* (min)	FOC*^ a^* (Patient/Control)	*P*-value*^ b^*	VIP*^ c^*	Chemical class	AUC (95% CI) *^d^*	*P*-value*^ d^*
1	Pyroglutamate^*^	C_5_H_7_NO_3_	129.043	4.974	-7.524	<0.001	1.202	Amino acids	0.873 (0.801 - 0.944)	<0.001
2	Methionine^*^	C_5_H_11_NO_2_S	149.051	0.952	-24.955	<0.001	1.380	Amino acids	0.685 (0.557 - 0.793)	0.006
3	5-acetamidovalerate^*^	C_7_H_14_NO_3_	159.089	3.486	-15.543	<0.001	1.282	Amino acids	0.762 (0.647 - 0.877)	<0.001
4	S-(2-carboxypropyl)-Cysteamine^#^	C_6_H_13_NO_2_S	163.066	1.356	-36.231	<0.001	1.204	Amino acids	0.510 (0.394 - 0.625)	0.886
5	Methylhistidine^#^	C_7_H_11_N_3_O_2_	169.085	0.566	-4.146	<0.001	0.834	Amino acids	0.897 (0.835 - 0.959)	<0.001
6	N-lactoyl-Valine^#^	C_8_H_15_NO_4_	189.100	4.603	-17.175	<0.001	0.952	Amino acids	0.837 (0.744 - 0.931)	<0.001
7	N-Acetylaminooctanoic acid^#^	C_10_H_19_NO_3_	201.137	8.886	-5.477	<0.001	0.973	Amino acids	0.777 (0.675 - 0.879)	<0.001
8	N-lactoyl-Leucine^#^	C_10_H_13_N_5_	203.117	0.693	14.632	0.020	0.959	Amino acids	0.749 (0.634- 0.864)	<0.001
9	4-Hydroxy-3-methoxy-cinnamoylglycine^#^	C_12_H_13_NO_5_	251.080	5.811	-2552.980	<0.001	2.664	Amino acids	0.926 (0.847 - 1.000)	<0.001
10	Alpha-N-Phenylacetyl-L-glutamine^*^	C_13_H_16_N_2_O_4_	264.111	4.974	-2.196	<0.001	0.837	Amino acids	0.773 (0.678 - 0.868)	<0.001
11	N-Acetylleucine ^#^	C_8_H_15_NO_3_	173.106	5.638	-2.170	<0.001	0.614	Amino acids	0.705 (0.596 - 0.814)	0.002
12	Indoleacetic acid^#^	C_10_H_9_NO_2_	175.064	8.188	-6.239	<0.001	0.845	Amino acids	0.677 (0.562 - 0.791)	0.009
13	Octenoylglycine^#^	C_10_H_17_NO_3_	199.121	8.452	-2.474	0.032	0.524	Amino acids	0.562 (0.434 - 0.691)	0.357
14	N-Propionylmethionine^#^	C_8_H_15_NO_3_S	205.077	4.66	9.583	0.014	1.098	Amino acids	0.588 (0.461 - 0.715)	0.193
15	N-Acetyltryptophan^#^	C_13_H_14_N_2_O_3_	246.101	7.509	9.136	0.019	0.879	Amino acids	0.518 (0.380 - 0.655)	0.793
16	Pyro-L-glutaminyl-L-glutamine^#^	C_10_H_15_N_3_O_5_	257.101	1.026	-4.299	<0.001	0.874	Amino acids	0.510 (0.390 - 0.629)	0.886
17	Hydroxyphenylacetylglycine^#^	C_10_H_11_NO_4_	209.069	6.013	-119.931	<0.001	1.638	Amino acids	0.758 (0.652 - 0.863)	<0.001
18	Hepteneoylglycine^#^	C_9_H_15_NO_3_	185.105	6.357	-1730.717	<0.001	2.480	Amino acids	0.783 (0.653 - 0.912)	<0.001
19	Creatinine^*^	C_4_H_7_N_3_O	113.059	0.645	2.851	0.018	0.441	Amino acids	0.542 (0.411 - 0.672)	0.537
20	Indolylacryloylglycine^#^	C_13_H_12_N_2_O_3_	244.086	7.857	-30.454	<0.001	1.476	Amino acids	0.774 (0.658 - 0.889)	<0.001
21	8-Hydroxy-5,6-octadienoic acid^#^	C8H12O3	156.079	6.806	-6.225	0.005	1.219	Fatty acids	0.519 (0.393 - 0.644)	0.780
22	N-Heptanoylglycine^#^	C_9_H_17_NO_3_	187.122	6.183	1194.673	<0.001	2.493	Fatty acids	0.932 (0.884 - 0.980)	<0.001
23	cis-4-Decenedioic acid^#^	C_10_H_16_O_4_	200.105	6.285	-9.210	<0.001	1.083	Fatty acids	0.717 (0.173 - 0.394)	<0.001
24	Alanylasparagine^#^	C_9_H_18_NO_4_	203.116	6.515	-77.434	<0.001	1.468	Fatty acids	0.842 (0.763 - 0.922)	<0.001
25	1-Methyl-2-nonyl-4(1H)-quinolinone^#^	C_15_H_27_NO_4_	285.193	7.987	-3.594	0.016	0.929	Quinolones and derivatives	0.609 (0.474 - 0.744)	0.108
26	3,4-Methylenesebacic acid^#^	C_12_H_18_O_4_	226.122	7.755	-17.150	<0.001	1.437	Fatty acids	0.700 (0.576 - 0.824)	0.003
27	2-trans,4-cis-Decadienoylcarnitine^#^	C_17_H_29_NO_4_	311.210	8.867	-2.488	<0.001	0.833	Fatty acids	0.702 (0.588 - 0.816)	0.003
28	4-Hydroxy-(3',4'-dihydroxyphenyl)-valeric acid^#^	C_11_H_14_O_5_	226.121	8.219	-4.113	0.001	0.937	Fatty acids	0.642 (0.526 - 0.758)	0.035
29	N^1^,N^12^-Diacetylspermine^#^	C_14_H_30_N_4_O_2_	286.238	0.614	277.128	<0.001	1.990	Carboximidic acids	0.819 (0.717 - 0.921)	<0.001
30	Hippurate^*^	C_9_H_9_NO_3_	179.059	4.841	-4.755	<0.001	1.069	Benzoic acids	0.830 (0.745 - 0.914)	<0.001
31	Hydroxyhippurate^#^	C_9_H_9_NO_4_	195.054	3.621	-12497.060	<0.001	3.005	Benzoic acids	0.865 (0.767 - 0.963)	<0.001
32	Prolyl-Valine^#^	C_10_H_18_N_2_O_3_	214.131	0.996	-6.156	0.008	0.938	Dipeptide	0.817 (0.718 - 0.915)	<0.001
33	Aspartylphenylalanine^#^	C_13_H_16_N_2_O_5_	280.106	4.230	-10.818	<0.001	1.131	Dipeptide	0.828 (0.733 - 0.923)	<0.001
34	Phenylacetylglutamine^#^	C_8_H_13_N_3_O_6_	264.108	4.740	14.025	0.014	1.070	Amino acids	0.636 (0.506 - 0.766)	0.044
35	Humulinic acid A	C_13_H_18_N_2_O_4_	266.124	7.507	-2572.774	<0.001	2.499	Dipeptide	0.862 (0.773 - 0.950)	<0.001
36	Ubiquinone-1^#^	C_14_H_18_O_4_	250.120	7.813	-8.768	<0.001	1.142	Quinone	0.693 (0.568 - 0.818)	0.004
37	4-Pyridoxic acid^#^	C_8_H_9_NO_4_	183.053	1.394	4.161	<0.001	0.656	Pyridinecar-boxylic acids	0.662 (0.553 - 0.771)	0.016
38	alpha-D-Glucose^#^	C_10_H_12_O_3_	180.079	6.907	-11.393	<0.001	1.249	Carbohydrates	0.629 (0.502 - 0.756)	0.056
39	3-Hydroxydodecanedioic acid^#^	C_12_H_22_O_5_	246.149	8.070	-19.079	<0.001	1.174	Hydroxy acids	0.774 (0.673 - 0.874)	<0.001
40	Indoxyl^#^	C_8_H_7_NO	133.053	4.795	-3.872	0.002	0.871	Indoxyl	0.749 (0.650 - 0.849)	<0.001
41	Glutamylproline^#^	C_9_H_12_N_2_O_6_	244.070	0.944	-2.031	0.010	0.454	Amino acids	0.613 (0.496 - 0.730)	0.094

*^a^*FOC was calculated from the arithmetic mean values. FOC with a positive value means a relative higher concentration in CRC patients, while a negative value indicates a relative lower concentration as compared to controls. *^b^P* value was calculated from student's t test. *^c^*Variable importance in the project (VIP) was obtained from OPLS-DA. *^d^*AUC and *p* value was obtained by ROC curve analysis on the basis of the training set. Abbreviations: FOC, Fold of changes. AUC, area under the curve. These metabolites were identified by commercially approached standards (^*^) or web-based spectrum databases (^#^).

**Table 2:** Differential metabolites related to capecitabine related AE

Metabolites	HFS			Anemia			Neutropenia			Thrombocytopenia			BMS		
	FOC*^a^*	AUC (95% CI)*^b^*	*P*-value*^b^*	FOC*^a^*	AUC (95% CI)*^b^*	*P-*value*^b^*	FOC*^a^*	AUC (95% CI)*^b^*	*P*-value*^b^*	FOC*^a^*	AUC (95% CI)*^b^*	*P-*value*^b^*	FOC*^a^*	AUC (95% CI)*^b^*	*P-*value*^b^*
Methionine	-1.738	0.731 (0.569-0.893)	0.029^*^	-1.208	0.612 (0.418-0.807)	0.281	-1.268	0.592 (0.398-0.785)	0.355	1.083	0.543 (0.347-0.739)	0.665	-1.198	0.557 (0.354-0.759)	0.594
5-acetamidovalerate	-1.603	0.716 (0.554-0.878)	0.042^*^	-3.994	0.902 (0.784-1.000)	<0.001^*^	-1.620	0.719 (0.546-0.891)	0.027^*^	1.117	0.553 (0.359-0.748)	0.594	-1.382	0.625 (0.438-0.812)	0.241
Methylhistidine	1.182	0.541 (0.330-0.751)	0.701	-1.595	0.754 (0.551-0.956)	0.015^*^	-1.477	0.650 (0.465-0.836)	0.129	1.342	0.573 (0.381-0.766)	0.463	1.011	0.545 (0.342-0.749)	0.670
N-Acetylaminooctanoic acid	-1.926	0.631 (0.451-0.811)	0.215	-1.781	0.732 (0.548-0.915)	0.026^*^	1.049	0.510 (0.309-0.711)	0.921	1.172	0.633 (0.447-0.820)	0.182	-1.185	0.511 (0.308-0.715)	0.915
N-Acetylleucine	-1.174	0.531 (0.304-0.759)	0.768	-2.266	0.583 (0.391-0.775)	0.424	-1.405	0.797 (0.652-0.943)	0.003^*^	-1.125	0.590 (0.398-0.782)	0.368	-1.529	0.674 (0.488-0.861)	0.102
Indoleacetic acid	-1.326	0.625 (0.425-0.825)	0.238	-1.616	0.786 (0.601-0.971)	0.006^*^	4.286	0.598 (0.402-0.794)	0.322	2.210	0.513 (0.315-0.712)	0.894	2.618	0.655 (0.467-0.843)	0.145
1-Methyl-2-nonyl-4(1H)-quinolinone	-1.773	0.684 (0.497-0.872)	0.081	-2.907	0.841 (0.631-1.000)	0.001^*^	-2.017	0.752 (0.585-0.918)	0.011^*^	-1.310	0.543 (0.349-0.738)	0.665	-1.880	0.693 (0.518-0.868)	0.070
Hydroxyphenylacetylglycine	1.816	0.647 (0.441-0.853)	0.165	6.386	0.507 (0.281-0.734)	0.945	3.181	0.667 (0.483-0.850)	0.092	2.637	0.700 (0.526-0.874)	0.046^*^	2.192	0.670 (0.493-0.848)	0.110
Creatinine	-1.219	0.575 (0.368-0.782)	0.478	-1.662	0.707 (0.521-0.892)	0.048^*^	-1.530	0.709 (0.533-0.885)	0.035^*^	-1.125	0.690 (0.511-0.869)	0.057	-1.308	0.705 (0.504-0.905)	0.055
Indolylacryloylglycine	-7.358	0.716 (0.539-0.892)	0.042^*^	-2.399	0.667 (0.466-0.867)	0.110	-2.125	0.552 (0.357-0.748)	0.597	-1.153	0.567 (0.363-0.770)	0.505	-1.409	0.595 (0.369-0.820)	0.374
3,4-Methylenesebacic acid	-1.343	0.634 (0.418-0.851)	0.204	-3.602	0.826 (0.681-0.972)	0.002^*^	-1.969	0.752 (0.587-0.916)	0.011^*^	-1.259	0.547 (0.352-0.741)	0.641	-1.808	0.674 (0.493-0.856)	0.102
2-trans,4-cis-Decadienoylcarnitine	-1.488	0.678 (0.490-0.867)	0.092	-1.705	0.703 (0.503-0.903)	0.052	-1.775	0.716 (0.540-0.891)	0.029^*^	-1.064	0.530 (0.332-0.728)	0.764	-1.395	0.580 (0.383-0.776)	0.456
N^1^,N^12^-Diacetylspermine	-2.605	0.731 (0.501-0.962)	0.029^*^	1.557	0.500 (0.298-0.702)	1.000	1.073	0.578 (0.386-0.771)	0.428	-2.621	0.563 (0.365-0.762)	0.527	-1.256	0.561 (0.352-0.770)	0.570
Hippurate	-3.304	0.65 (0.451-0.849)	0.156	-2.456	0.710 (0.524-0.896)	0.044^*^	-1.737	0.526 (0.326-0.726)	0.792	-1.507	0.507 (0.310-0.703)	0.947	-1.997	0.591 (0.391-0.79)	0.394
Aspartylphenylalanine	-1.377	0.619 (0.404-0.834)	0.262	-1.924	0.717 (0.518-0.916)	0.037^*^	-1.224	0.565 (0.372-0.758)	0.509	-1.141	0.543 (0.350-0.737)	0.665	-1.331	0.580 (0.382-0.777)	0.456
Phenylacetylglutamine	-1.421	0.684 (0.497-0.872)	0.081	-1.938	0.841 (0.631-1.000)	0.001^*^	-1.619	0.752 (0.585-0.918)	0.011^*^	-1.402	0.543 (0.349-0.738)	0.665	-1.689	0.693 (0.518-0.868)	0.070
Ubiquinone-1	-1.862	0.684 (0.471-0.897)	0.081	-3.688	0.793 (0.617-0.970)	0.005^*^	-2.499	0.771 (0.609-0.933)	0.006^*^	-1.613	0.590 (0.386-0.794)	0.368	-2.238	0.689 (0.488-0.891)	0.076
4-Pyridoxic acid	3.222	0.744 (0.550-0.938)	0.021^*^	2.362	0.754 (0.592-0.915)	0.015^*^	-1.350	0.546 (0.342-0.750)	0.644	-1.082	0.590 (0.384-0.796)	0.368	1.936	0.739 (0.529-0.948)	0.025^*^
Indoxyl	-1.141	0.675 (0.500-0.850)	0.098	-1.553	0.859 (0.663-1.000)	0.001^*^	-1.455	0.637 (0.446-0.828)	0.166	-1.473	0.553 (0.358-0.749)	0.594	-1.928	0.614 (0.425-0.803)	0.286

*^a^*FOC was calculated from the arithmetic mean values. FOC with a positive value means a relative higher concentration in CRC patients, while a negative value indicates a relative lower concentration as compared to controls. *^b^*AUC and *p* value was obtained by ROC curve analysis. ^*^P-value < 0.5 is considered to have statistical significance. Abbreviations: FOC, Fold of changes. AUC, area under the curve.

**Table 3:** Metabolic pathway enrichment analysis based on colorectal cancer related metabolites

Pathway	Source	External_id	*P*-value	Matched metabolite
Glucose Homeostasis	Wikipathways	WP661	0.0000	Methionine, Hippurate, D-Glucose
Vitamin B12 Metabolism	Wikipathways	WP1533	0.0004	Methionine, Creatinine, D-Glucose
Conjugation of carboxylic acids	Reactome	R-HSA-159424	0.0008	Hippurate, Alpha-N-Phenylacetyl-L-glutamine,
Amino Acid conjugation	Reactome	R-HSA-156587	0.0008	Hippurate, Alpha-N-Phenylacetyl-L-glutamine,
Trans-sulfuration pathway	Wikipathways	WP4253	0.0031	Methionine, Creatinine,
Mineral absorption - Homo sapiens (human)	KEGG	path:hsa04978	0.0036	Methionine, D-Glucose,
Amino Acid metabolism	Wikipathways	WP3925	0.0039	Methionine, Indoleacetic acid, D-Glucose
Phase II - Conjugation of compounds	Reactome	R-HSA-156580	0.0057	Pyroglutamate, Hippurate, Alpha-N-Phenylacetyl-L-glutamine
Central carbon metabolism in cancer - Homo sapiens (human)	KEGG	path:hsa05230	0.0058	Methionine, D-Glucose,
One carbon metabolism and related pathways	Wikipathways	WP3940	0.0074	Pyroglutamate, Methionine,
Folate Metabolism	Wikipathways	WP176	0.0108	Methionine, D-Glucose,
Phenylalanine metabolism - Homo sapiens (human)	KEGG	path:hsa00360	0.0209	Hippurate, Alpha-N-Phenylacetyl-L-glutamine,
Tryptophan metabolism - Homo sapiens (human)	KEGG	path:hsa00380	0.0261	Indoleacetic acid, Indoxyl,
Selenium Micronutrient Network	Wikipathways	WP15	0.0279	Methionine, D-Glucose,
Biological oxidations	Reactome	R-HSA-211859	0.0403	Pyroglutamate, Hippurate, Alpha-N-Phenylacetyl-L-glutamine

Metabolic pathway enrichment analysis was carried by an on-line tool (CPDB, http://cpdb.molgen.mpg.de/). The pathway databases for matching included REACTOME, KEGG, SMPDB, and Wikipathways. The minimum overlap with input list was set as 2 and the p-value cutoff was set as 0.05.

**Table 4:** Metabolic pathway enrichment analysis based on capecitabine-related-adverse-effect related metabolites

Pathway	Source	External_id	*P*-value	Matched metabolite
Conjugation of carboxylic acids	Reactome	R-HSA-159424	0.0007	Hippurate, Alpha-N-Phenylacetyl-L-glutamine,
Amino Acid conjugation	Reactome	R-HSA-156587	0.0007	Hippurate, Alpha-N-Phenylacetyl-L-glutamine,
Glucose Homeostasis	Wikipathways	WP661	0.0016	Methionine, Hippurate,
Trans-sulfuration pathway	Wikipathways	WP4253	0.0027	Methionine, Creatinine,
Vitamin B12 Metabolism	Wikipathways	WP1533	0.0077	Methionine, Creatinine,
Phenylalanine metabolism - Homo sapiens (human)	KEGG	path:hsa00360	0.0181	Hippurate, Alpha-N-Phenylacetyl-L-glutamine,
Arginine and proline metabolism - Homo sapiens (human)	KEGG	path:hsa00330	0.0211	Creatinine, 4-Acetamidobutanoic acid,
Tryptophan metabolism - Homo sapiens (human)	KEGG	path:hsa00380	0.0226	Indoleacetic acid, Indoxyl,
Amino Acid metabolism	Wikipathways	WP3925	0.0367	Methionine, Indoleacetic acid,
Phase II - Conjugation of compounds	Reactome	R-HSA-156580	0.0468	Hippurate, Alpha-N-Phenylacetyl-L-glutamine,

Metabolic pathway enrichment analysis was carried by an on-line tool (CPDB, http://cpdb.molgen.mpg.de/). The pathway databases for matching included REACTOME, KEGG, SMPDB, and Wikipathways. The minimum overlap with input list was set as 2 and the p-value cutoff was set as 0.05.

## Abbreviations

AE: adverse effect
AUC: area under curve
BMS: bone marrow suppression
CA19-9: carbohydrate antigen 19-9
CEA: carcinoembryonic antigen
CRC: colorectal cancer
FOC: fold of change
HFS: hand foot syndrome
MSEA: Metabolic Sets Enrichment Analysis
OPLS-DA: orthogonal partial least squares discriminant analysis
ROC: receiver operating characteristic
RT: retention time
PCA: principal component analysis
QC: quality control
UHPLC-Q-TOF-MS: ultra-high-performance liquid chromatography combined with quadrupole time-of-flight mass spectrometry
VIP: importance in the projection
