# Supplementary material for: Profiling of polar urine metabolite extracts from Chinese colorectal cancer patients to screen for potential diagnostic and adverse-effect biomarkers
Source: J Cancer. 2020 Oct 8;11(23):6925–38. doi: 10.7150/jca.47631 (PMC7592006; doi:10.7150/jca.47631)
Supplement: Supplementary file 1 — Supplementary figures and tables. [file jcav11p6925s1.pdf]

Supplementary Data for

Profiling of polar urine metabolite extracts from Chinese colorectal cancer patients to screen for potential diagnostic and adverse-effect biomarker

Yi Deng [1#], Houshan Yao[2#], Wei Chen[1], Hua Wei[1], Xinxing Li[2], Feng Zhang[1], Shouhong Gao[1], Huan Man[1,3], Jing Chen[1,3], Xia Tao[1], Mingming Li[1], Wansheng Chen[1,4*]*

[1]Department of Pharmacy, Changzheng Hospital, Secondary Military Medical University, Shanghai, China

[2]Department of Surgery, Changzheng Hospital, Secondary Military Medical University, Shanghai, China

[3]College of Chemical and Biological Engineering, Yichun University, Jiangxi Province, China

[4] Research and Development Center of Chinese Medicine Resources and Biotechnology, Shanghai University of Traditional Chinese Medicine, Shanghai, China

[*]Corresponding Author:

Mingming Li, Department of Pharmacy, Changzheng Hospital, Secondary Military Medical University, Shanghai, China. Email:

limingming@smmu.edu.cn

Wansheng Chen, Department of Pharmacy, Changzheng Hospital, Secondary Military Medical University, Shanghai, China. Email:

chenwansheng@smmu.edu.cn.

[#]Equal first author

1. Supplementary Tables

Table S1. Clinical information for CRC patients and nonneoplastic controls

Clinical information	CRC patients	Nonneoplastic controls	*P*-value
In total			
Number	139	50	
Age (media, range)	63, 36 - 87	61, 47 - 89	0.234[a]
Male/female ratio	91/48	28/22	0.373[b]
TNM-0	8		
TNM-I	26		
TNM-II	42		
TNM-III	50		
TNM-IV	13		
Training set			
Number	70	25	
Age (media, range)	60, 36 - 87	60, 47 - 78	0.993[a]
Male/female ratio	46/24	16/9	0.877[b]
TNM-0	5		
TNM-I	13		
TNM-II	16		
TNM-III	32		
TNM-IV	4		
Testing set			

Number	69	25	
Age (media, range)	65, 40 - 87	61, 49 - 89	0.135[a]
Male/female ratio	45/24	12/13	0.131[b]
TNM-0	3		
TNM-I	13		
TNM-II	26		
TNM-III	18		
TNM-IV	9		

[a]Age differences between the two groups were analyzed by Student's *t* test. [b]Sex differences between the two groups were analyzed by Chi-square test.

Table S2. The grade of AEs in CRC patients with capecitabine-based adjuvant chemotherapy

Grade[a]	HFS	Bone marrow suppression			
		Anemia	Neutropenia	Thrombocytopenia	BMS
NA[b]	1	8	8	8	8
Grade 0	10	23	17	15	11
Grade 1-2	29	9	16	16	19
Grade 3-4	3	3	2	4	5

[a]Adverse effects were graded according to the Common Terminology Criteria for Adverse Events (Version 4.0). [b]These data were missing. Abbreviations: BMS, bone marrow suppression; HFS, hand foot syndrome.

Table S3. Clinical information of participants.

	Group	With AE	Without AE	*P*-value
**HFS**	Number	32	10	
	Age (media, range)	58, 37 - 74	57, 36 - 81	0.768[a]
	Male/female ratio	21/11	6/4	1.000[b]
	Cycles (media, range)	6.3, 1 - 8	6.5, 1 - 8	0.770[a]
	TNM-I	1	1	0.656[c]
	TNM-II	14	4	
	TNM-III	12	4	
	TNM-IV	5	1	
**Anemia**	Number	12	23	
	Age (media, range)	59, 49 - 74	57, 38 - 81	1.000[a]
	Male/female ratio	8/4	16/7	0.549[b]
	Cycles (media, range)	6.2, 2 - 8	6.7, 2 - 8	0.394[a]
	TNM-I	0	1	0.795[c]
	TNM-II	6	8	
	TNM-III	2	11	
	TNM-IV	4	3	
**Neutropenia**	Number	18	17	
	Age (media, range)	59, 37 - 74	57, 38 - 81	0.593[a]
	Male/female ratio	11/7	13/4	0.471[b]
	Cycles (media, range)	6.4, 2 - 8	6.7, 2 - 8	0.620[a]
	TNM-I	1	0	0.902[c]
	TNM-II	7	7	

	TNM-III	6	7	
	TNM-IV	4	3	
**Thrombocytopenia**	Number	20	15	
	Age (media, range)	59, 37 - 81	56, 38 - 68	0.329[a]
	Male/female ratio	13/7	11/4	0.721[b]
	Cycles (media, range)	7.0, 4 - 8	5.9, 2 - 8	0.092[a]
	TNM-I	1	0	0.845[c]
	TNM-II	7	7	
	TNM-III	9	4	
	TNM-IV	3	4	
**BMS**	Number	24	11	
	Age (media, range)	60, 37 - 81	54, 38 - 69	0.101[a]
	Male/female ratio	17/7	7/4	0.937[b]
	Cycles (media, range)	6.7, 2 - 8	6.3, 2 - 8	0.567[a]
	TNM-I	1	0	0.559[c]
	TNM-II	9	5	
	TNM-III	9	4	
	TNM-IV	5	2	

[a]Age difference and chemotherapy cycle was analyzed by Student's *t* test. [b]Sex difference was analyzed by Chi-square test. [c]Composition difference of the pathological stage was analyzed by Mann-Whitney test. Abbreviations: HFS: hand foot syndrome. BMS: bone marrow suppression.

Table S3. Updated mass spectrums of identified urine metabolites at first revision. For each metabolite identified by standard, the upper figure in each panel shows the spectrum from urine and lower panel shows the spectrum from commercial standards. Compound spectra 1, 2, 10, 19, 30 are obtained by comparison of our experiments with standards. The other metabolites are shown by the spectrum comparison results in HMDB.

No.	Metabolites	Mass spectrum of identified metabolites	Fit (%)	RFit (%)
1	Pyroglutamate	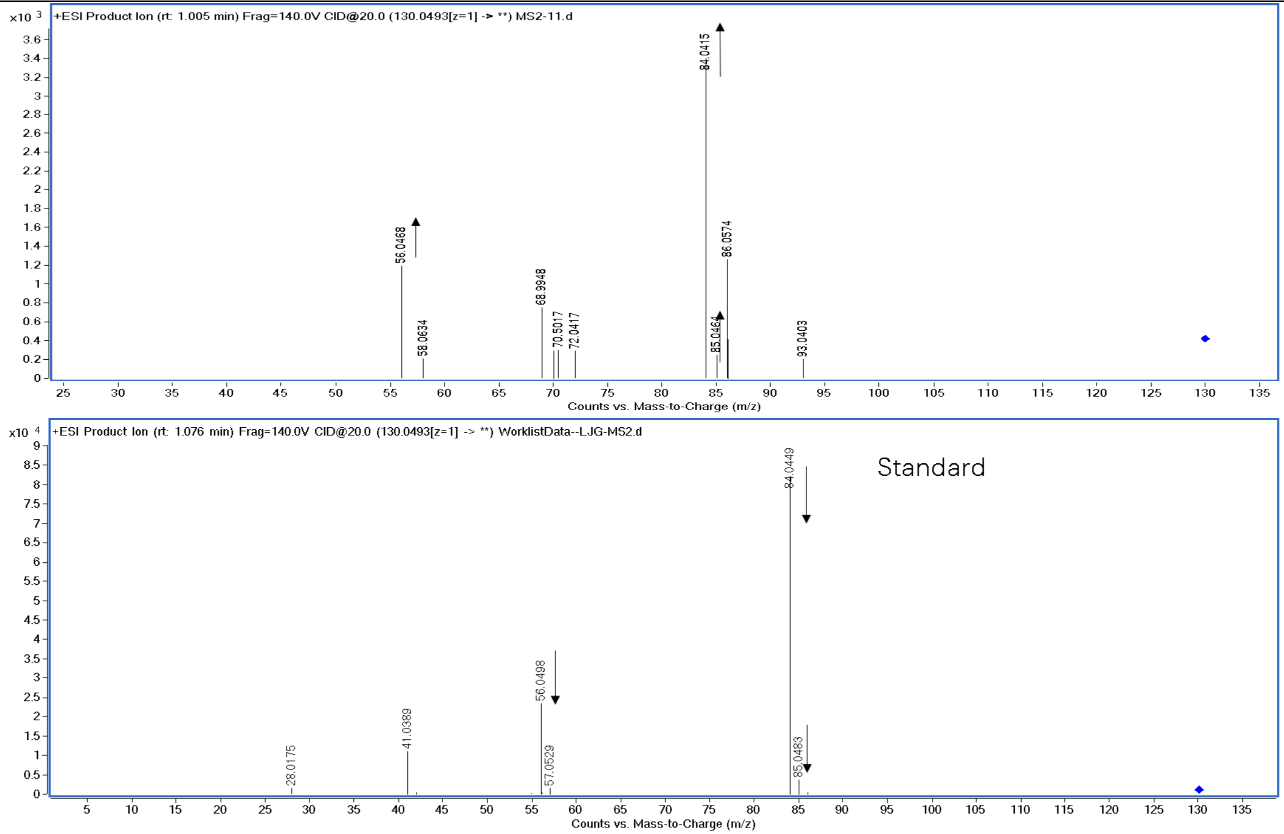	0.99	0.99

<table>
<tr><th></th><th></th><th></th><th></th><th></th></tr>
<tr><td>2</td><td>L-Methionine</td><td>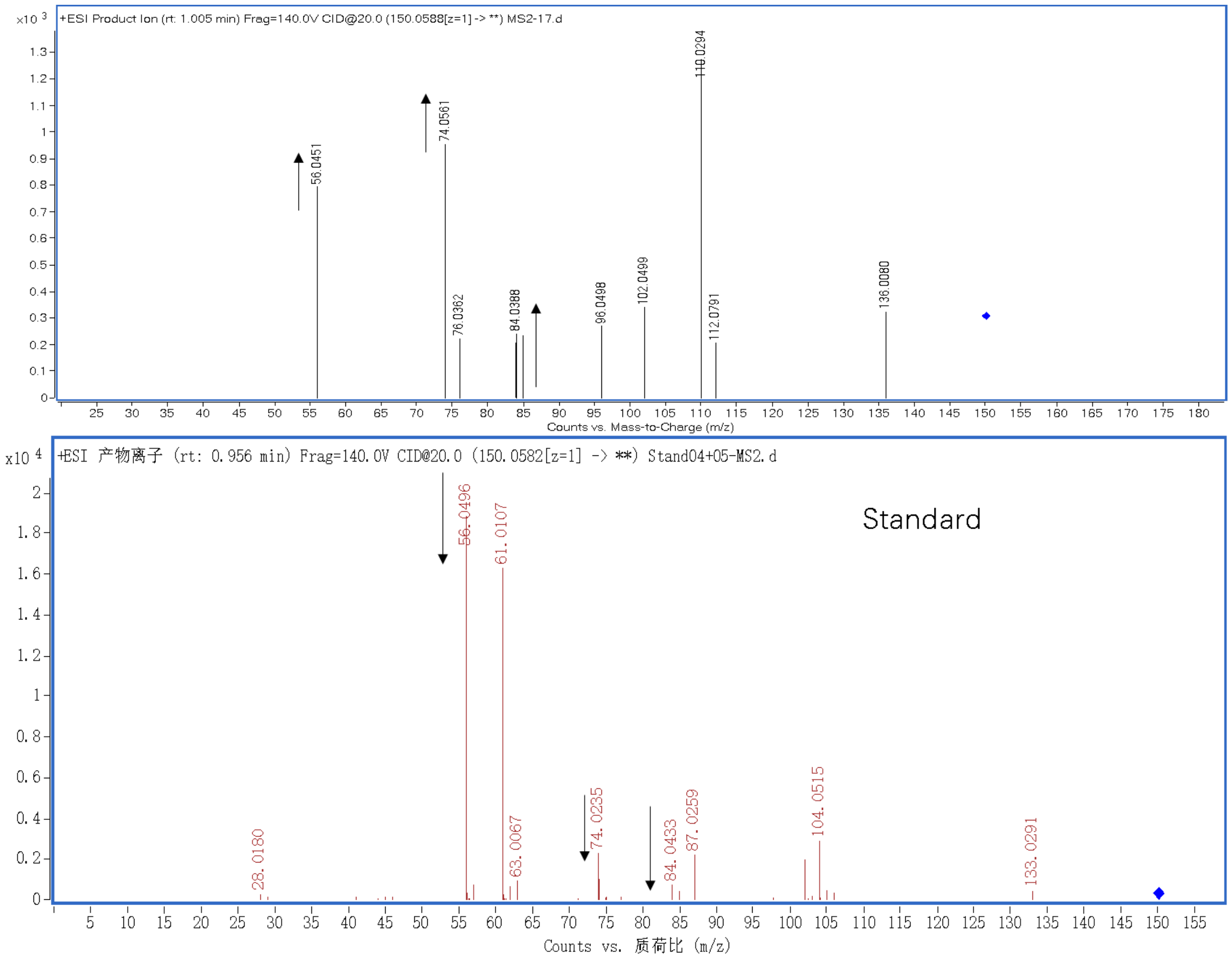
</td><td>0.99</td><td>0.99</td></tr>
</table>

3	5-Acetamidovalerate	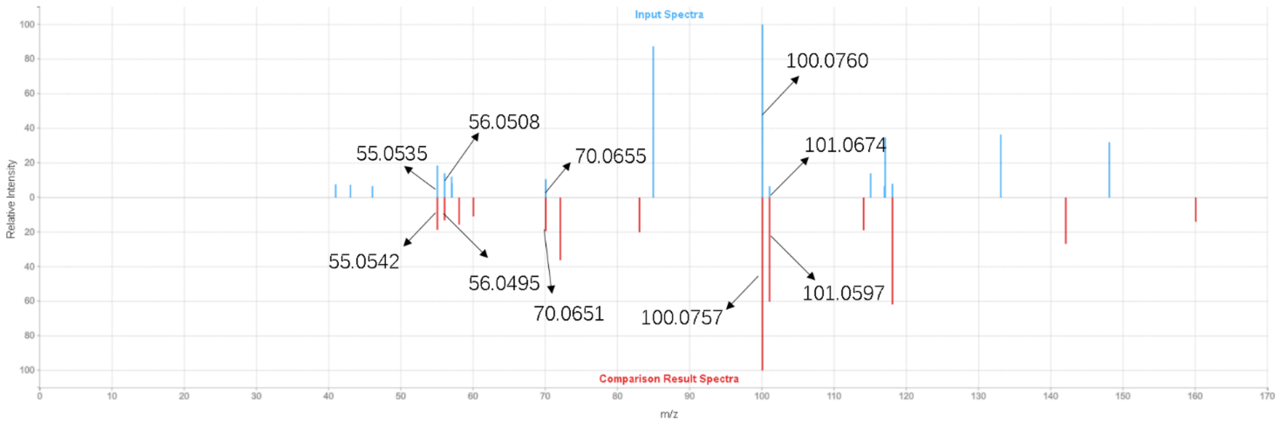	0.97	0.71
4	S-(2-carboxypropyl)-Cysteamine	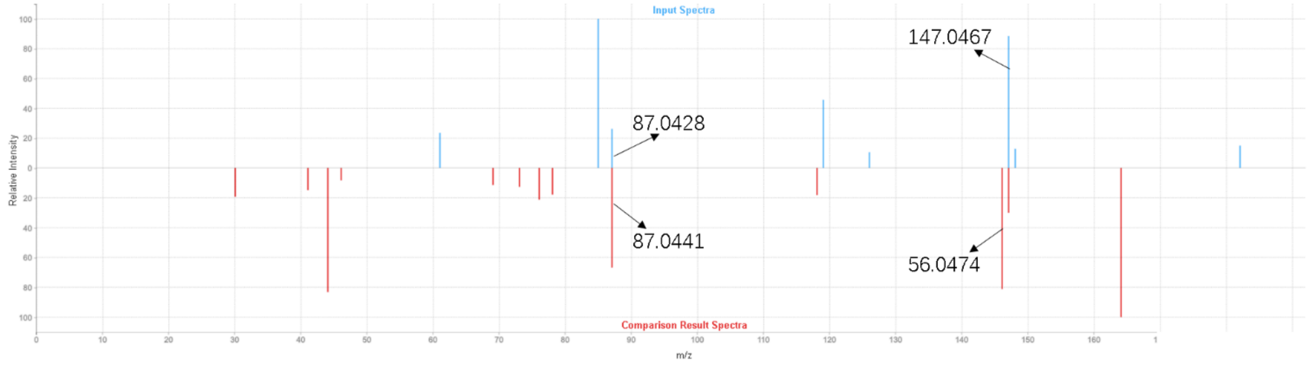	0.95	0.48

5	Methylhistidine	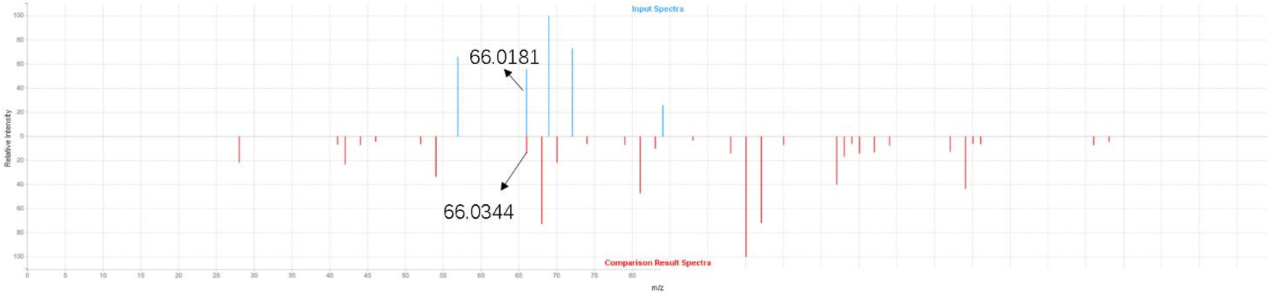	0.82	0.07
6	N-lactoyl-Valine	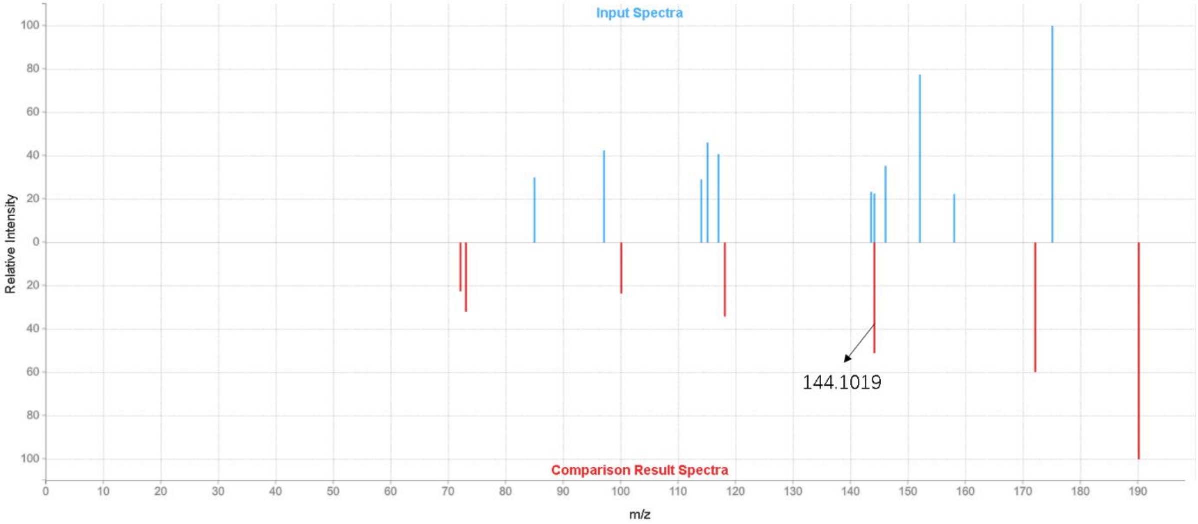	0.9	0.53

7	N-Acetylaminooctanoic acid	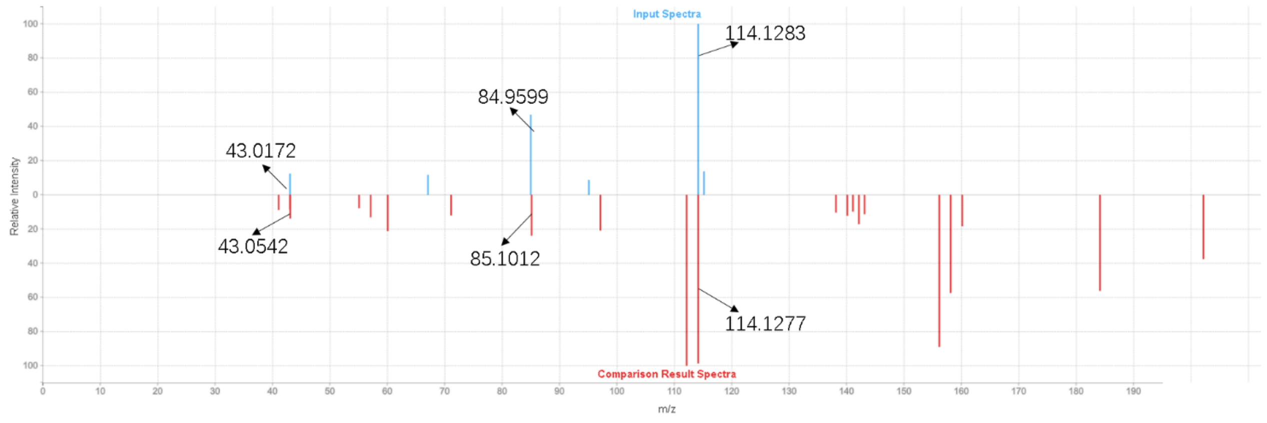	0.9	0.67
8	N-lactoyl-Leucine	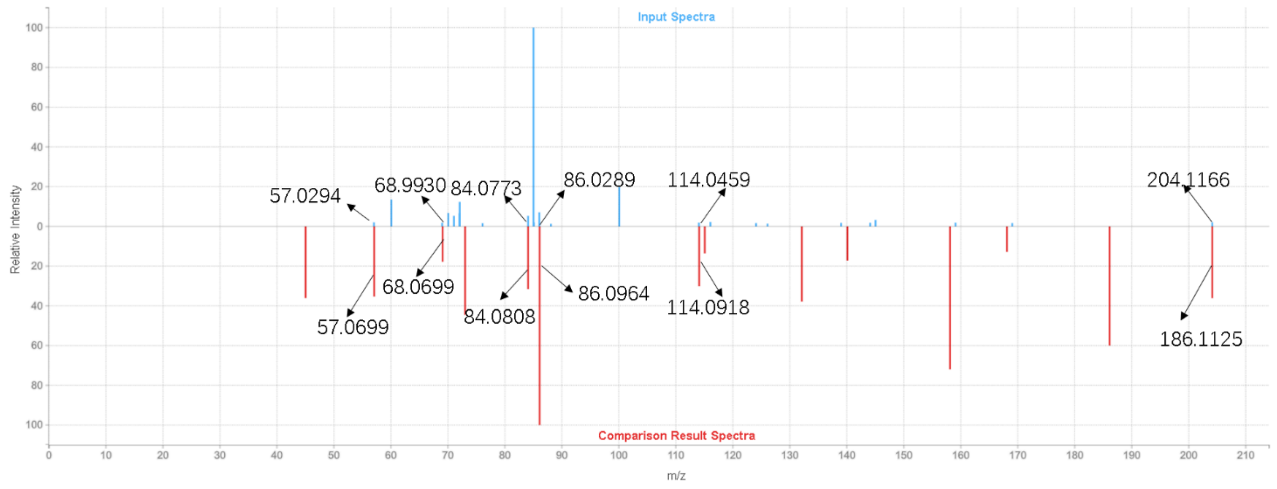	0.97	0.69

9	4-Hydroxy-3-methoxy-cinnamoylglycine	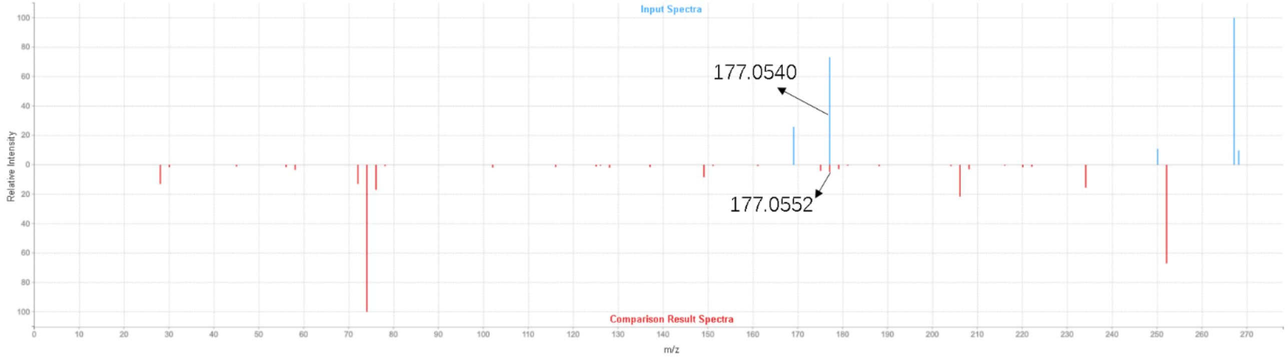	0.95	0.57

10	Alpha-N-Phenylacetyl-L-glutamine	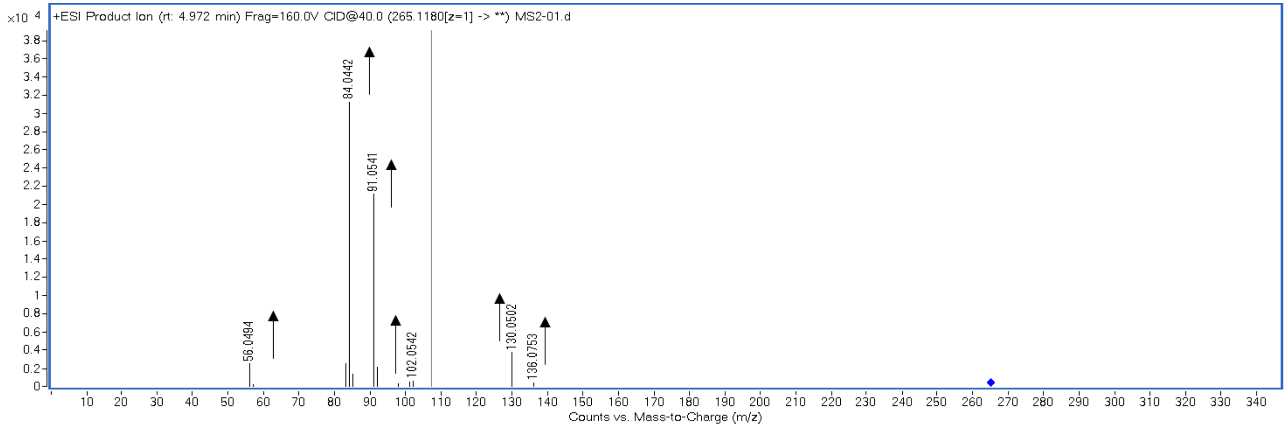 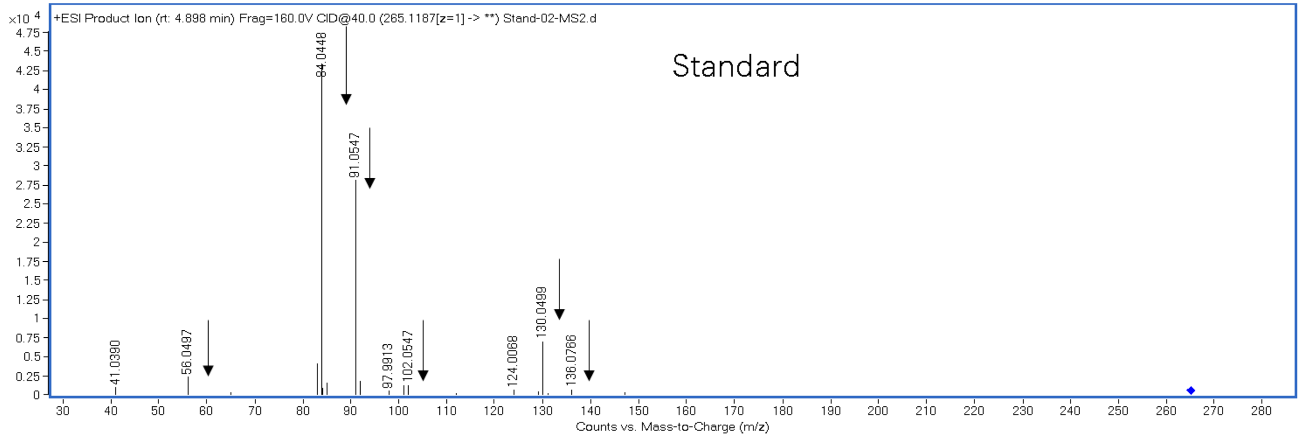	0.99	0.99

11	N-Acetylleucine	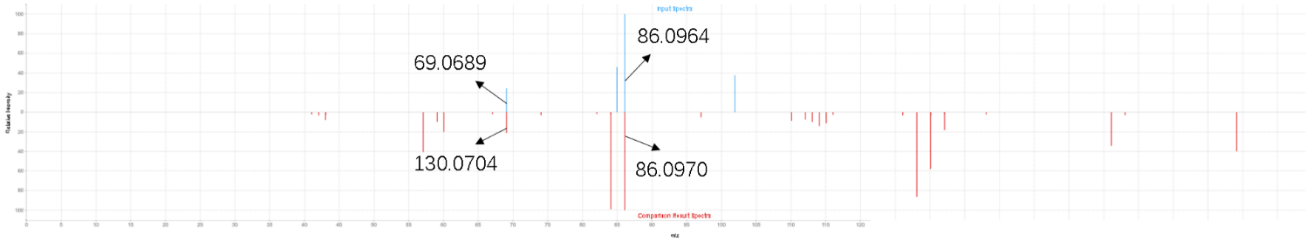	0.85	0.52
12	Indoleacetic acid	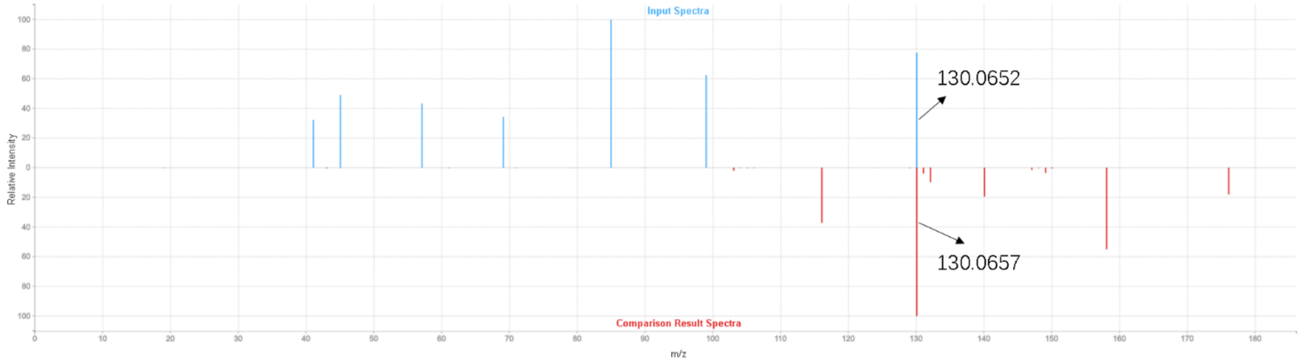	0.94	0.46

13	Octenoylglycine	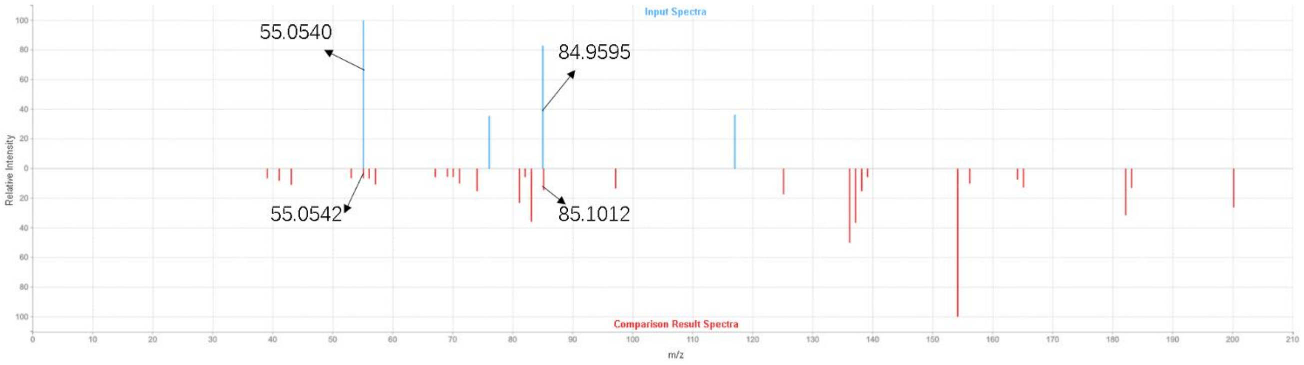	0.94	0.56
14	N-Propionylmethionine	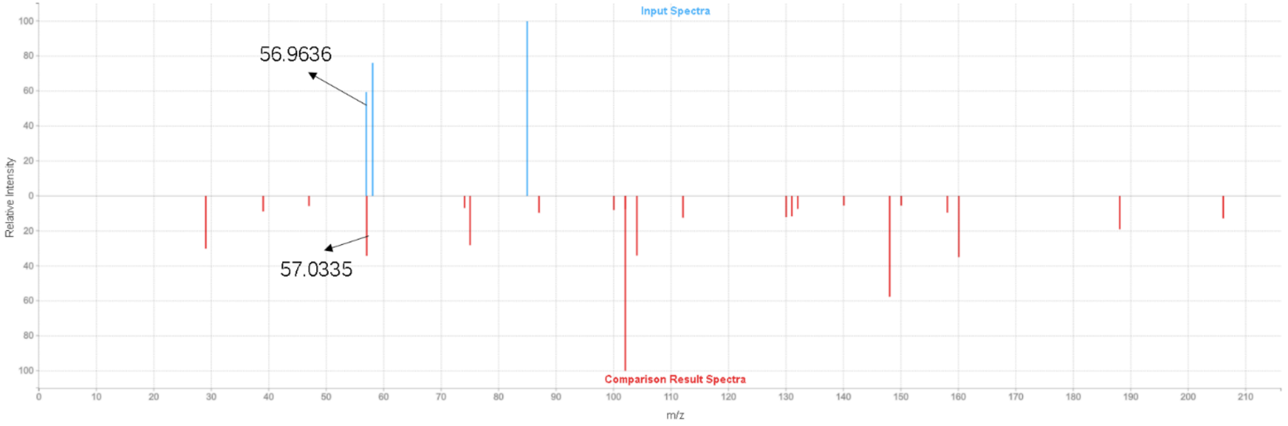	0.86	0.15

15	N-Acetyltryptophan	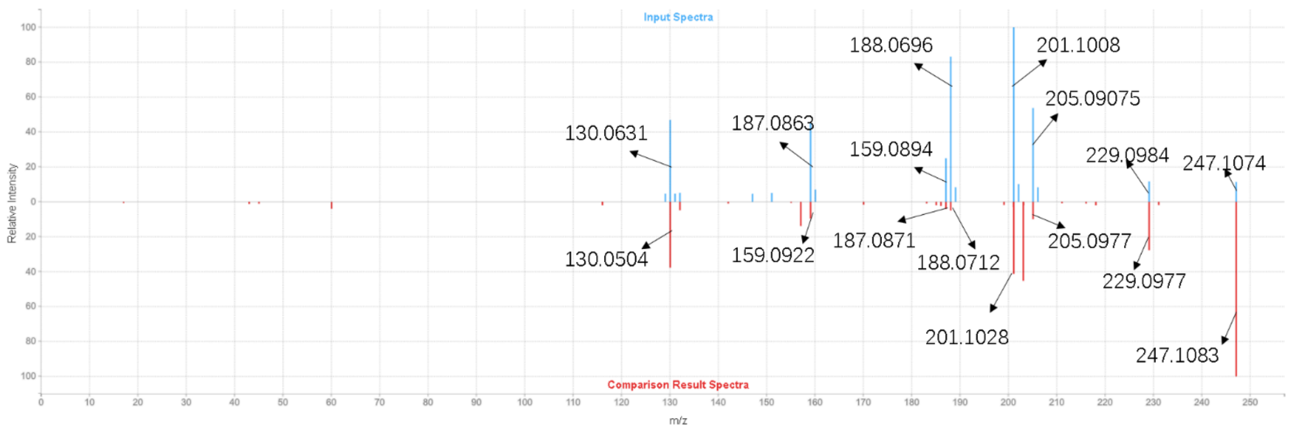	0.9	0.75
16	Pyro-L-glutaminyl-L-glutamine	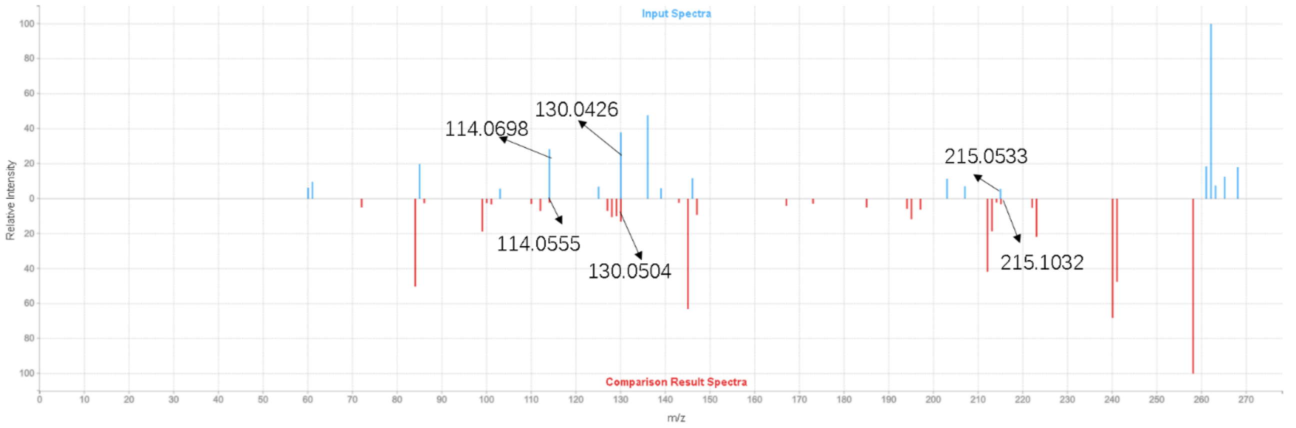	0.97	0.8

17	Hydroxyphenylacetylglycine	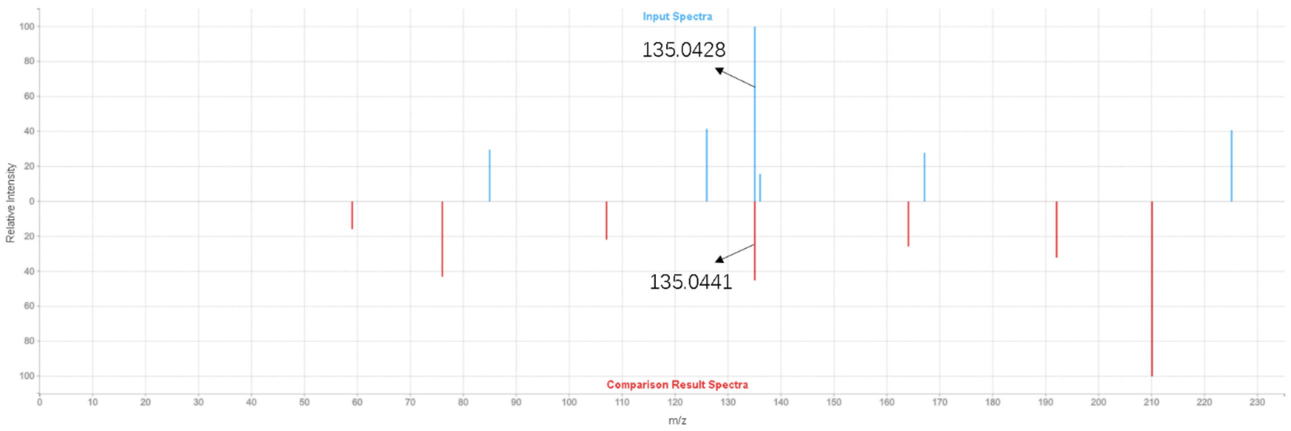	0.81	0.7
18	Hepteneoylglycine	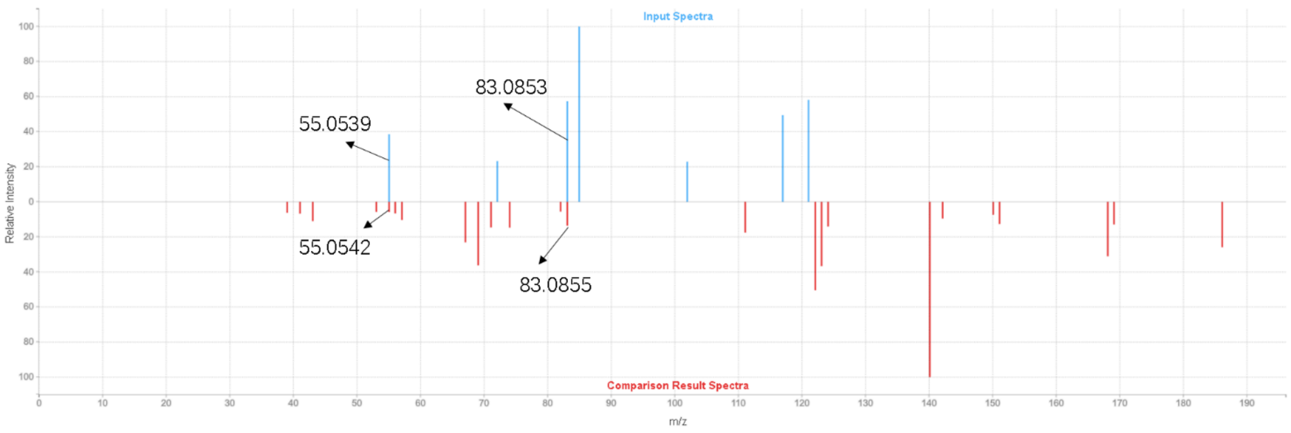	0.89	0.39

19	Creatinine	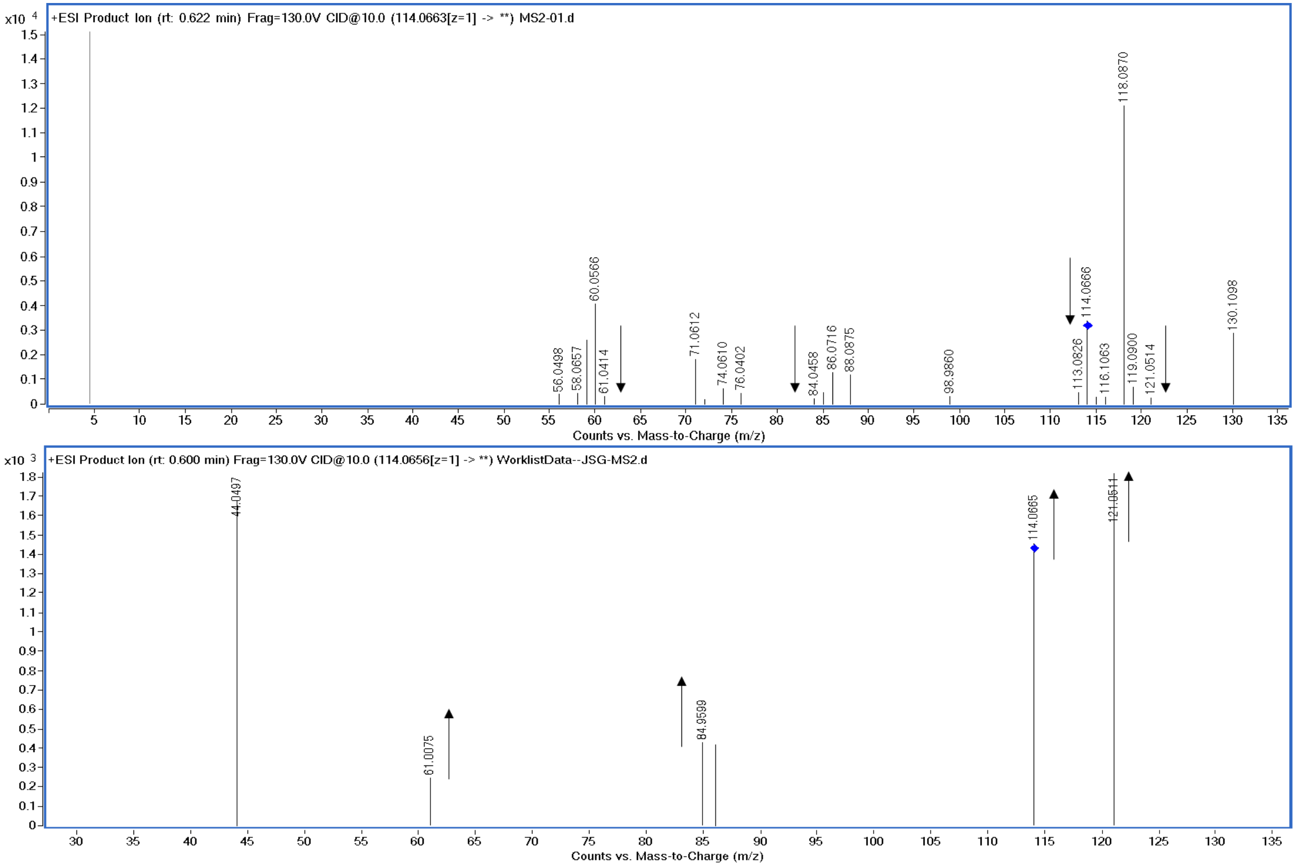	0.99	0.99

<table>
<tr><td>20</td><td>Indolylacryloylglycine</td><td>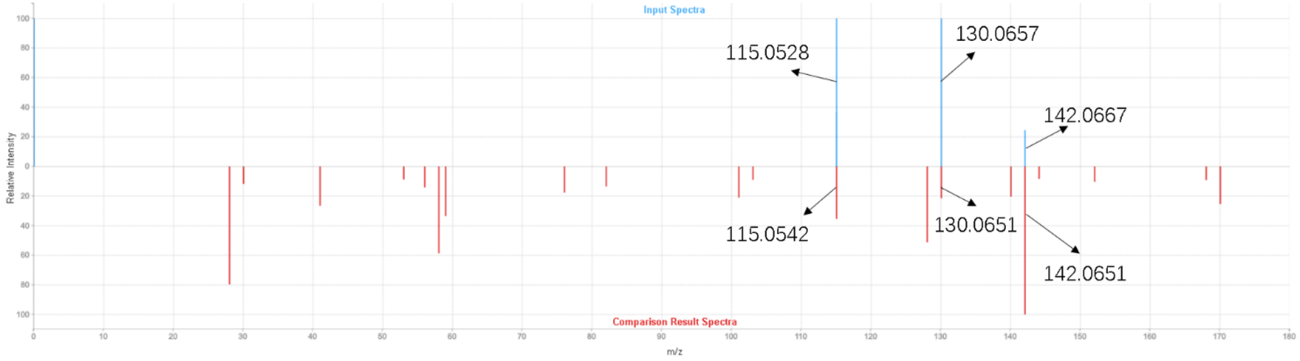
</td><td>0.92</td><td>0.68</td></tr>
<tr><td>21</td><td>8-Hydroxy-5,6-octadienoic</td><td>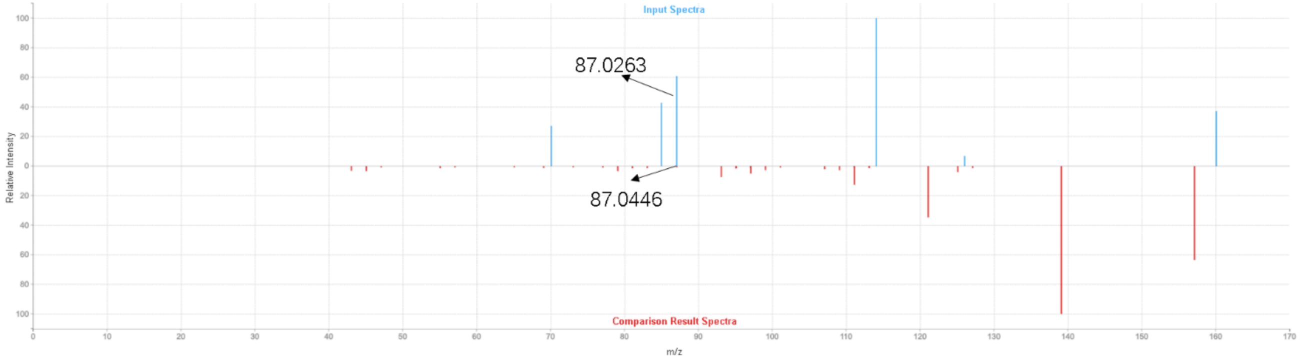
</td><td>0.84</td><td>0.49</td></tr>
</table>

22	N-Heptanoylglycine	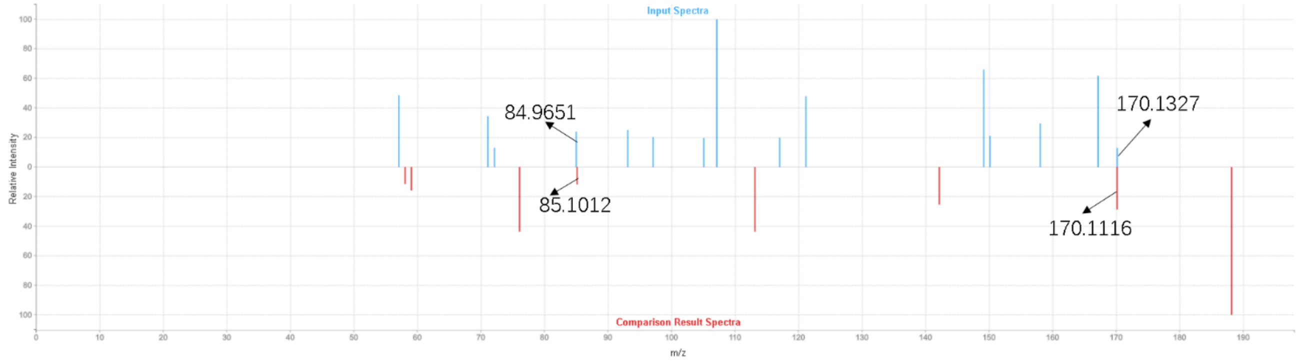	0.89	0.51
23	cis-4-Decenedioic acid	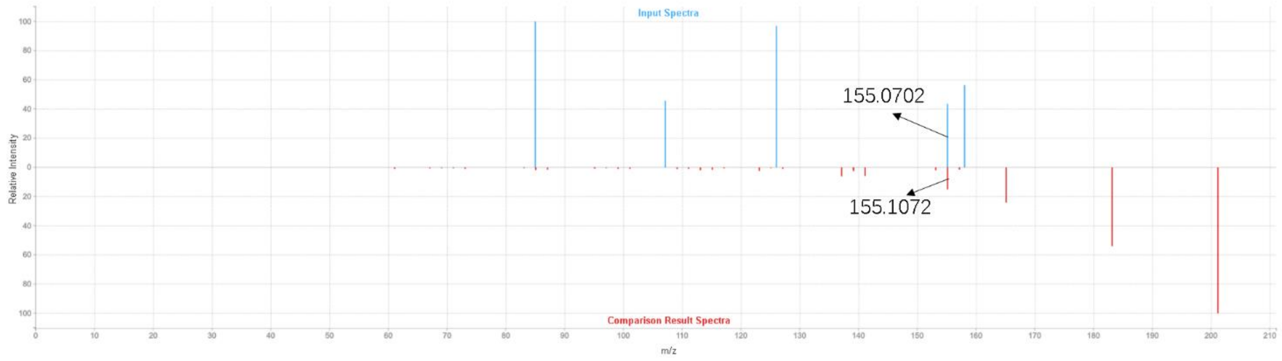	0.86	0.39

24	Alanylasparagine	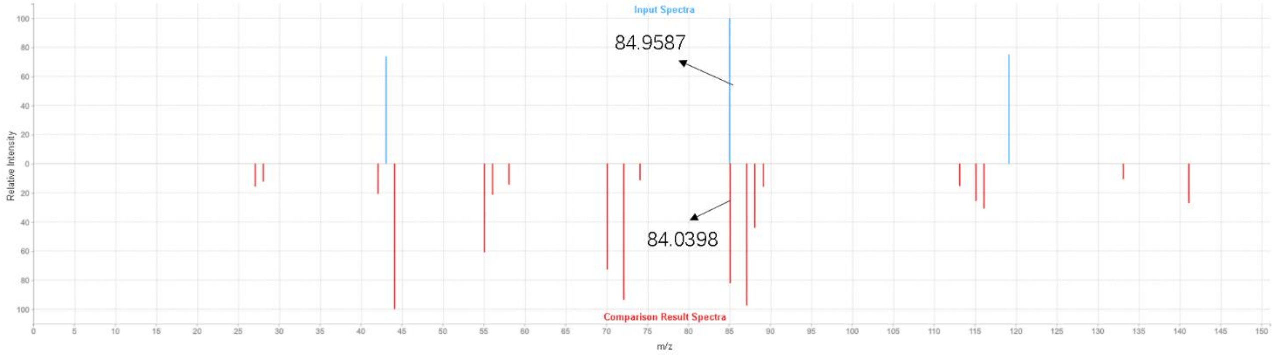	0.84	0.64
25	25 1-Methyl-2-nonyl-4(1H)-quinolinone	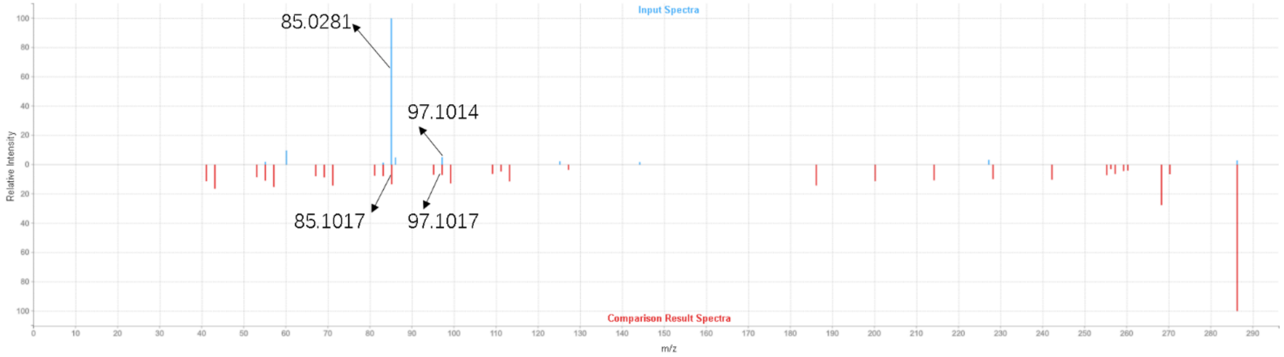	0.98	0.76

26	3,4-Methylenesebacic acid	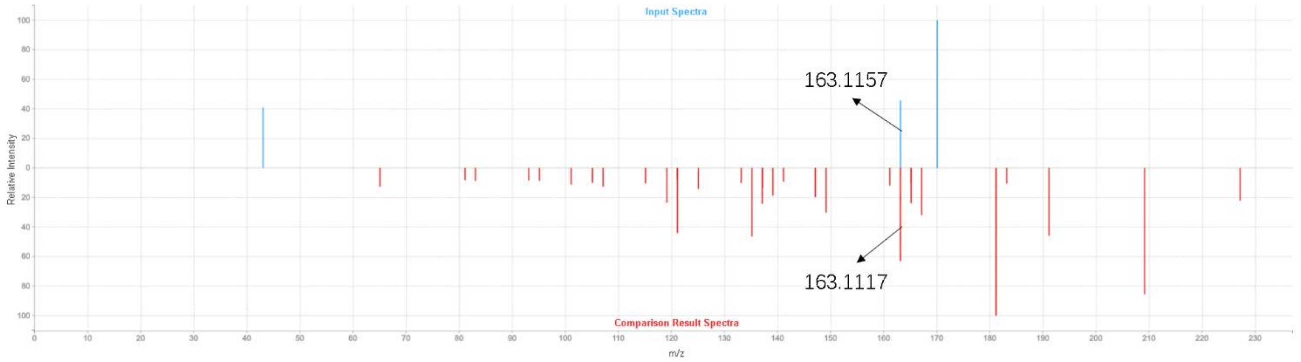	0.93	0.68
27	2-trans,4-cis-Decadienoylcarnitine	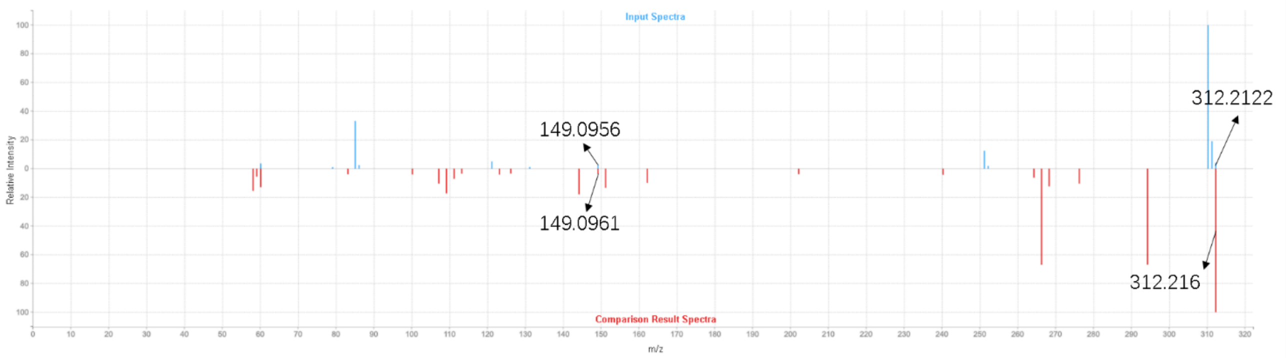	0.99	0.87

28	4-Hydroxy-(3',4'-dihydroxyphenyl)-valeric acid	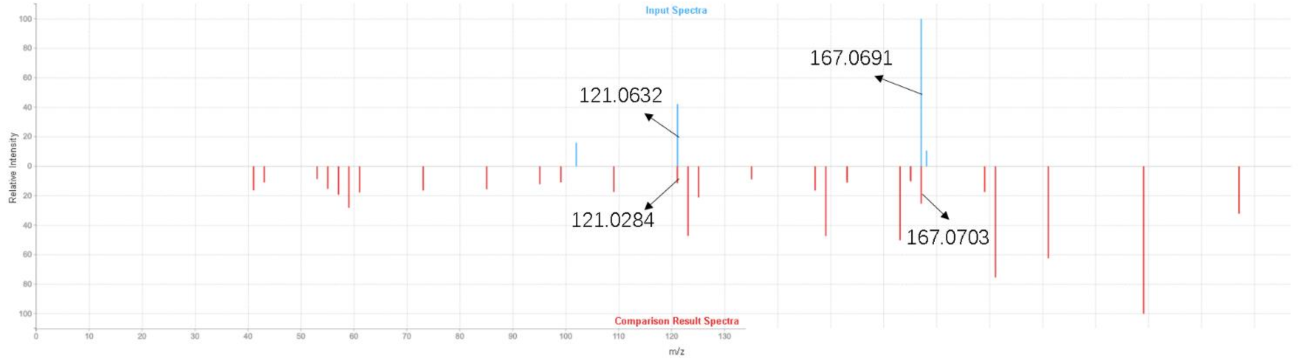	0.95	0.47
29	N^1N^{12}Diacetylspermine	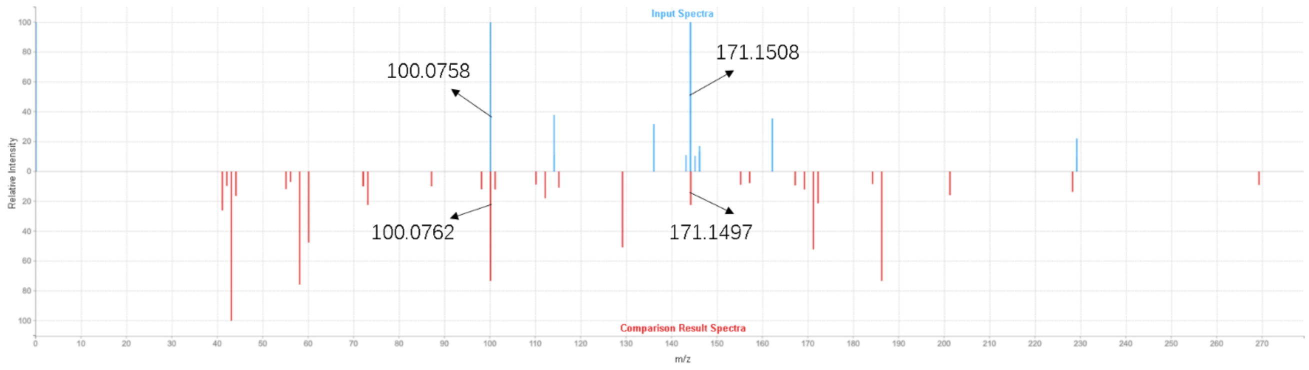	0.96	0.41

30	Hippurate	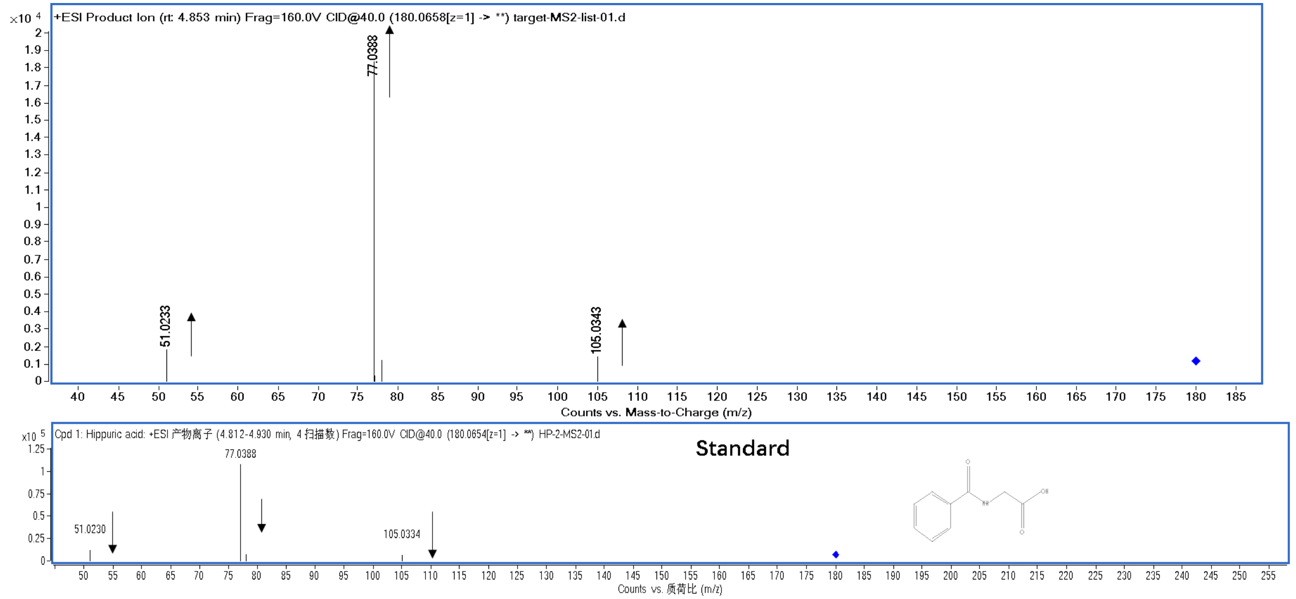	0.99	0.99
31	Hydroxyhippurate	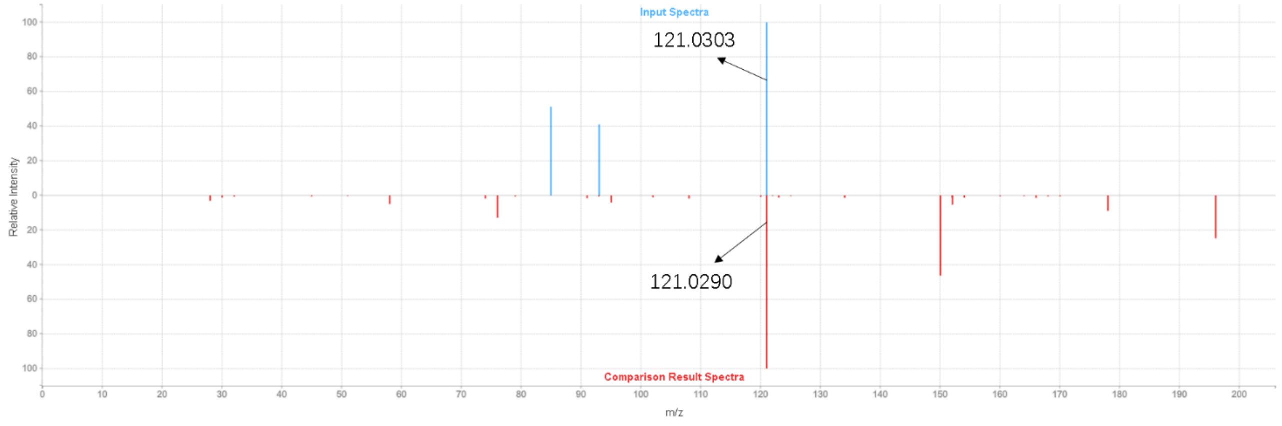	0.97	0.65

32	Prolyl-Valine	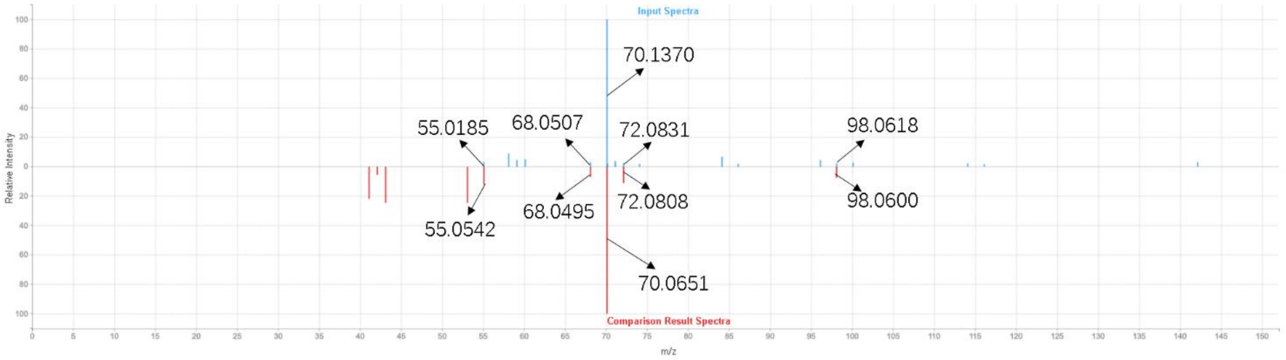	0.97	0.9
33	Aspartylphenylalanine	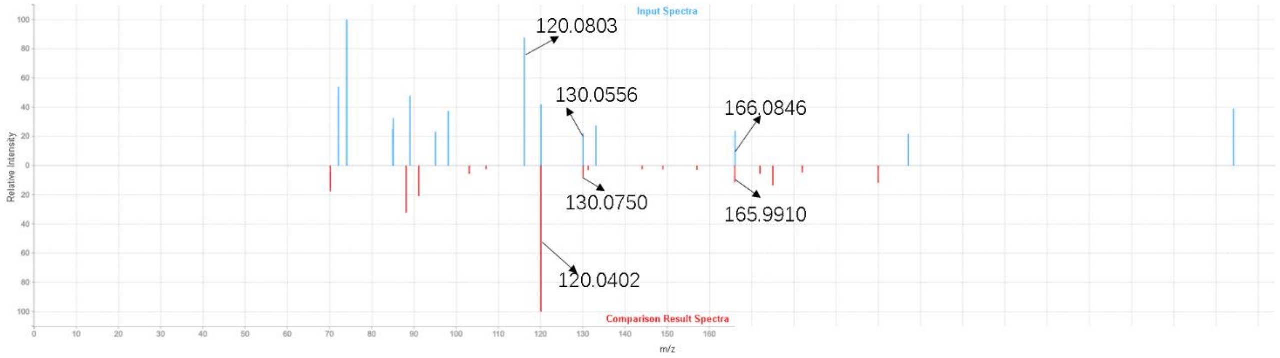	0.95	0.83

34	Phenylacetylglutamine	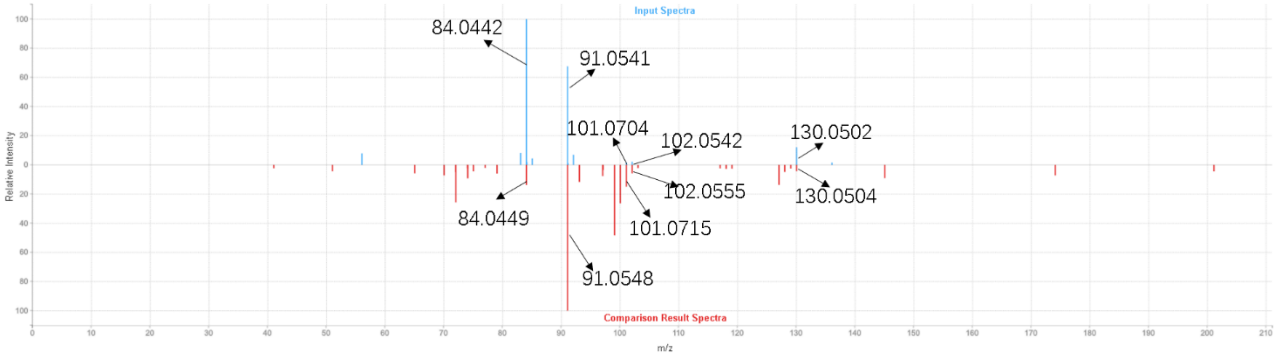	0.79	0.47
35	Humulinic acid A	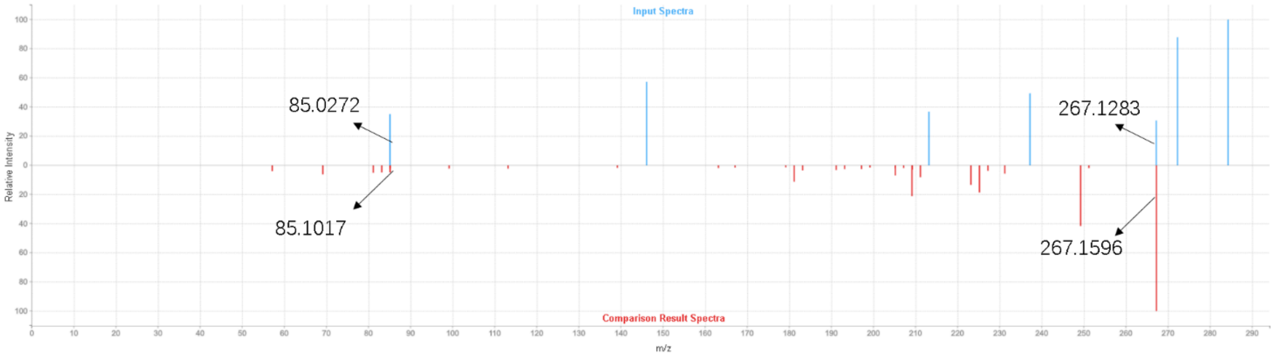	0.99	0.72

36	Ubiquinone-1	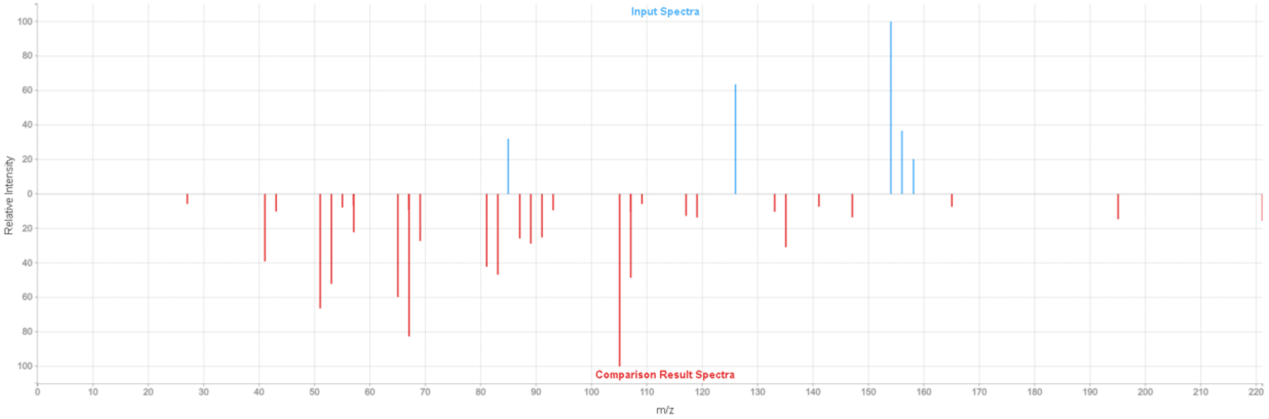	0.97	0.41
37	4-Pyridoxic acid	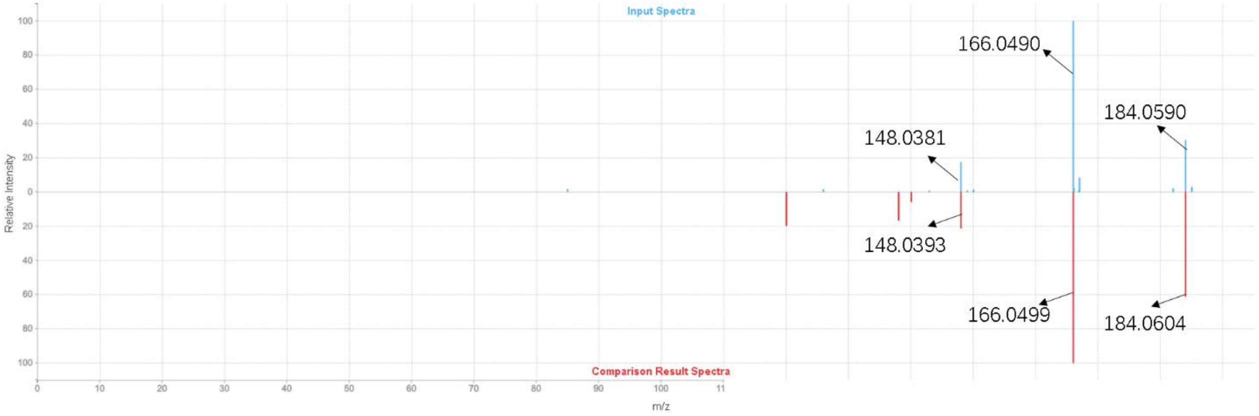	0.98	0.96

38	alpha-D-Glucose	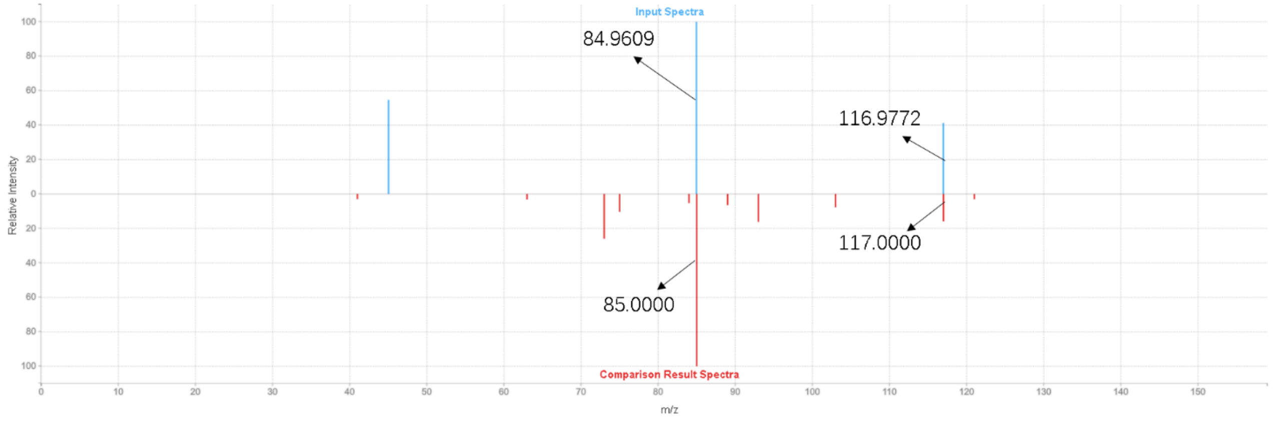	0.82	0.85
39	3-Hydroxydodecanedioic acid	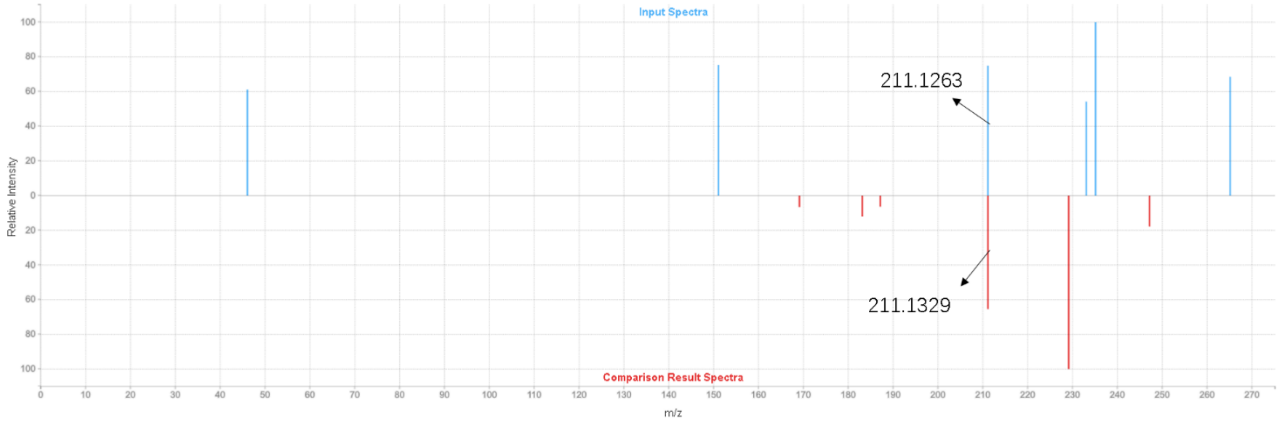	0.74	0.66

40	Indoxyl	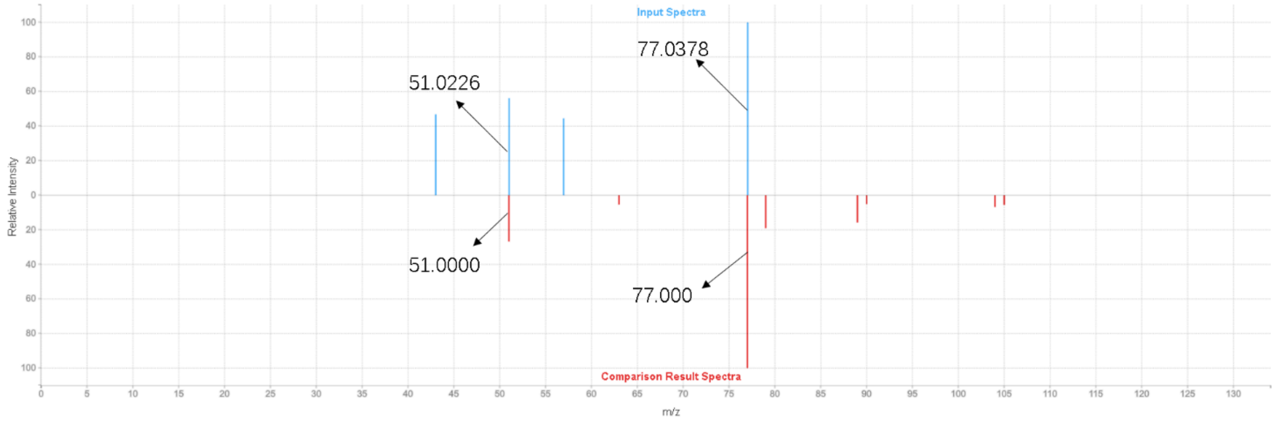	0.85	0.71
41	Glutamylproline	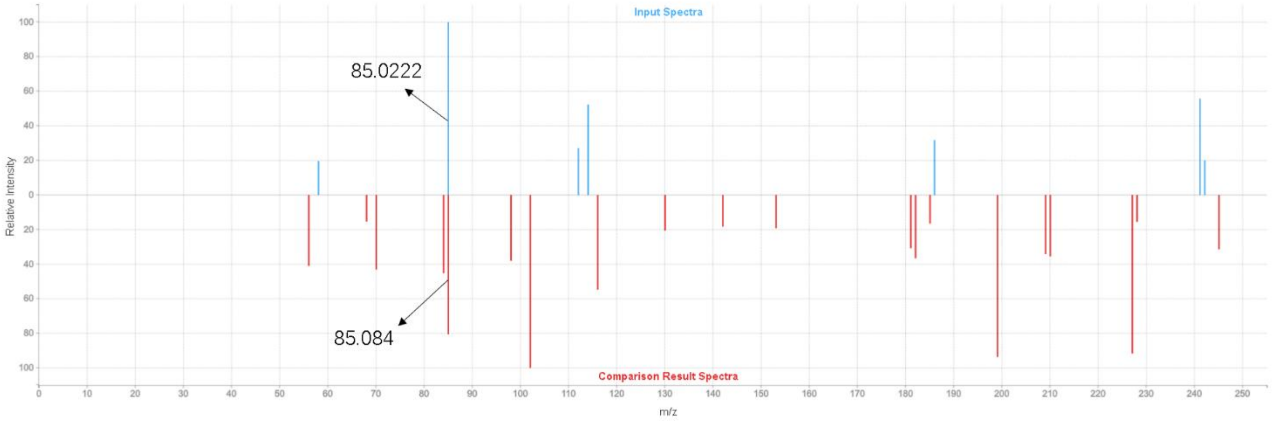	0.96	0.74

2. Supplementary Figures

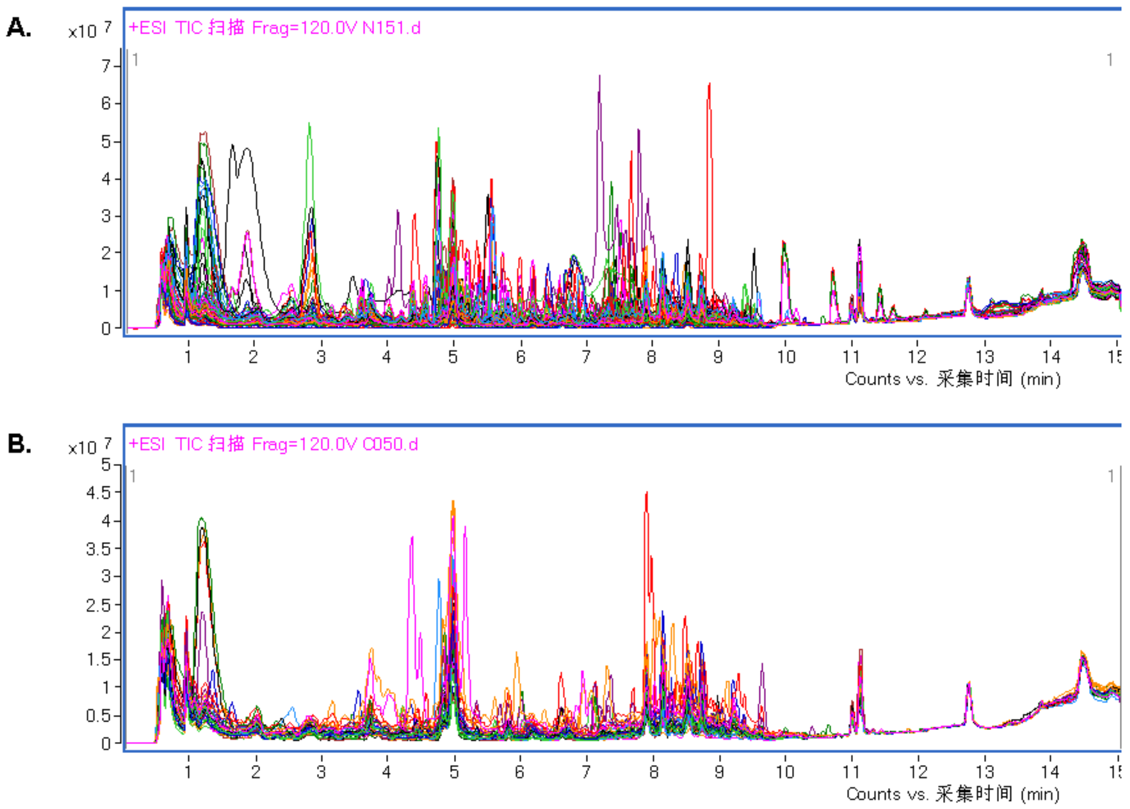

Fig. S1. Total ion chromatograms (TICs) of the urine metabolic profiles obtained from A1. CRC patients, B. nonneoplastic controls.

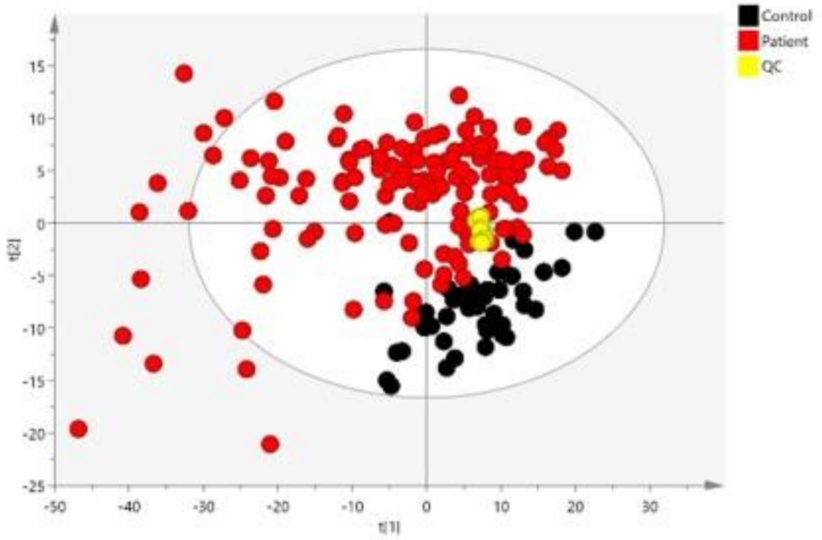

Component	R2X	R2X(cum)	Eigenvalue	Q2	Limit	Q2(cum)	Significance	Iterations
0	Cent.							
1	0.288	0.288	59.4	0.271	0.00657	0.271	R1	11
2	0.078	0.366	16.1	0.0916	0.00659	0.338	R1	26
3	0.0452	0.411	9.32	0.0523	0.00662	0.373	R1	28
4	0.0309	0.442	6.36	0.0228	0.00665	0.387	R1	73
5	0.0263	0.469	5.42	0.0151	0.00668	0.396	R1	125
6	0.025	0.494	5.14	0.007...	0.0067	0.401	R1	77
7	0.0222	0.516	4.58	0.0202	0.00673	0.413	R1	77
8	0.0195	0.535	4.02	0.008...	0.00676	0.418	R1	82
9	0.0179	0.553	3.68	0.0082	0.00679	0.422	R1	89
10	0.0155	0.569	3.2	0.005...	0.00682	0.425	R2	104
11	0.0145	0.583	2.98	-0.00...	0.00684	0.423	R2	97
12	0.0135	0.597	2.78	0.0007	0.00687	0.424	R2	100
13	0.0124	0.609	2.55	0.000...	0.0069	0.424	R2	200
14	0.0119	0.621	2.44	-0.00...	0.00693	0.423	R2	200
15	0.0114	0.632	2.35	-0.00...	0.00696	0.422	R2	81
16	0.0104	0.643	2.14	-0.00...	0.00699	0.419	R2	200
17	0.01	0.653	2.06	-0.00...	0.00702	0.419	R2	168

Fig. S2. Principal component analysis (PCA) of QC and experimental samples.

PCA plot (left) and the table of fitness scores (right) are presented. The PCA with all features indicates a tight clustering of QC samples. Black: QC samples, red: CRC patients, yellow: control patients.
